# Time course of bilateral microglial activation in a mouse model of laser-induced glaucoma

**DOI:** 10.1038/s41598-020-61848-9

**Published:** 2020-03-17

**Authors:** Ana I. Ramírez, Rosa de Hoz, José A. Fernández-Albarral, Elena Salobrar-Garcia, Blanca Rojas, Francisco J. Valiente-Soriano, Marcelino Avilés-Trigueros, María P. Villegas-Pérez, Manuel Vidal-Sanz, Alberto Triviño, José M. Ramírez, Juan J. Salazar

**Affiliations:** 1 Instituto de Investigaciones Oftalmológicas Ramón Castroviejo, Universidad Complutense de Madrid, Madrid, Spain; 2 Facultad de Óptica y Optometría, Departamento de Inmunología, Oftalmología y ORL, Universidad Complutense de Madrid, Madrid, Spain; 3 Facultad de Medicina, Departamento de Inmunología, Oftalmología y ORL, Universidad Complutense de Madrid, Madrid, Spain; 4 Departamento de Oftalmología, Facultad de Medicina, Universidad de Murcia and Instituto Murciano de Investigación Biosanitaria Virgen de la Arrixaca, Murcia, Spain

**Keywords:** Animal disease models, Microglia, Retina

## Abstract

Microglial activation is associated with glaucoma. In the model of unilateral laser-induced ocular hypertension (OHT), the time point at which the inflammatory process peaks remains unknown. Different time points (1, 3, 5, 8, and 15 d) were compared to analyze signs of microglial activation both in OHT and contralateral eyes. In both eyes, microglial activation was detected in all retinal layers at all time points analyzed, including: i) increase in the cell number in the outer segment photoreceptor layer and plexiform layers (only in OHT eyes) from 3 d onward; ii) increase in soma size from 1 d onward; iii) retraction of the processes from 1 d in OHT eyes and 3 d in contralateral eyes; iv) increase in the area of the retina occupied by Iba-1+ cells in the nerve fiber layer/ganglion cell layer from 1 d onward; v) increase in the number of vertical processes from 1 d in contralateral eyes and 3 d in OHT eyes. In OHT eyes at 24 h and 15 d, most Iba-1+ cells were P2RY12+ and were down-regulated at 3 and 5 d. In both eyes, microglial activation was stronger at 3 and 5 d (inflammation peaked in this model). These time points could be useful to identify factors implicated in the inflammatory process.

## Introduction

The neurodegenerative disease glaucoma is one of the main causes of irreversible blindness^1^. Standard treatments aim to control intraocular pressure (IOP), but this is often insufficient to prevent loss of visual field^2^. Many studies have attempted to clarify the pathogenic mechanisms in glaucoma, but it remains poorly understood. This multifactorial optical neuropathy is known to involve the progressive loss of retinal ganglion cells^3^.

Risk factors for glaucoma include elevated IOP^4^*,* vascular dysfunction, oxidative stress and immune-related neuroinflammation^5–10^. Another contributing factor is microglial activation, which helps drive inflammation that contributes to loss of retinal ganglion cells^11^. Such loss can be inhibited by blocking microglial activation with minocycline^12,13^, high-dose irradiation^14^ or gastrodin^15^.

Microglial cells are activated by an increase in IOP^16^, which causes their soma to enlarge and processes to retract. Activated microglia become migratory and begin to function as antigen-presenting cells and to proliferate^17^. These microglia express and secrete a range of inflammatory mediators into the retina and aqueous humor, including receptors, proteases, reactive oxygen species, cytokines and nitric oxide^18–21^. Activated microglia cells densely extend their processes toward the lesion site within a few minutes^22,23^, or they migrate to the site of damage^24,25^ to prevent lesion spread. These first responses are mediated by metabotropic purinergic P2Y12 receptors, which are stimulated by ATP released from dead or injured neurons^22,23^. P2RY12 is a specific marker for rodent resident microglial cells^26^ and is not expressed by monocytes or macrophages^27,28^. This chemotactic receptor regulates microglial activation^29^, and its signaling activity may enable microglia to respond to altered synaptic activity due to various types of injury^29^.

While activated microglial appear to contribute to neuronal death in glaucoma, they also express neuroprotective molecules and help remove debris created as retinal ganglion cells die^30^. Therefore, microglial activation needs to be analyzed carefully in order to gain new insights into the contribution of inflammation to the pathophysiology of this disease.

Our group took an initial step in this direction by examining microglial activation in a mouse model of unilateral laser-induced ocular hypertension (OHT) at 24 h^31^ and 15 d^32^ after laser induction. This microglial activation was observed in OHT and contralateral normotensive eyes, being more pronounced in OHT eyes. However, we are unaware of studies with this mouse model in which different time points after laser-induced OHT were compared and in which microglial activation was examined over time, in particular when activation peaked. Therefore, we made a comparative study of different signs of microglial activation (cell number, soma size, process retraction, number of vertical processes) at different time points (1, 3, 5, 8 and 15 days) in a mouse model of unilateral laser-induced OHT. We identified microglia based on the expression of not only Iba-1 but also P2RY12, allowing us to distinguish resident microglia from cells derived from infiltrating monocytes or macrophages during glaucoma-induced inflammation.

## Results

Microglial activation is characterized by soma enlargement, process retraction and increase in microglial number. These parameters were measured at various times after unilateral laser induction of OHT.

### IOP

**Figure 1:**
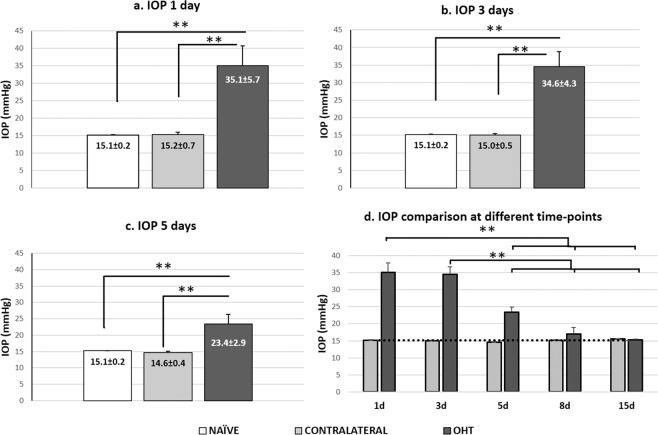
Measurement of intraocular pressure. (**a–d**) Histograms show mean (±SD) intraocular pressure (IOP) for naïve, contralateral and ocular hypertensive (OHT) eyes at 1 d (**a**), 3 d (**b**), 5 d (**c**). d: Variation in IOP over time in OHT and contralateral eyes. The dotted line indicates mean IOP in naïve eyes. In OHT eyes, pressure rose and peaked at 1 and 3 d, remained stable at 5 d and then gradually returned to basal preoperative levels by 8 d. Contralateral eyes showed similar pressures as naïve eyes at all time points. *p < 0.05, **p < 0.01.

IOP in laser-treated eyes showed the maximum difference from naïve eyes at 1 and 3 d after treatment (both p < 0.01; Fig. 1a,b,d). By 5 d, pressure began to fall but was still significantly higher than in naïve eyes (p < 0.01; Fig. 1c,d); by 8 d, pressure in laser-treated eyes was similar to that in naïve eyes (Fig. d). At all time points, contralateral eyes had similar IOP as naïve eyes (p > 0.05; Fig. 1a–d).

### Number of Iba-1**+** cells in the OS

**Figure 2:**
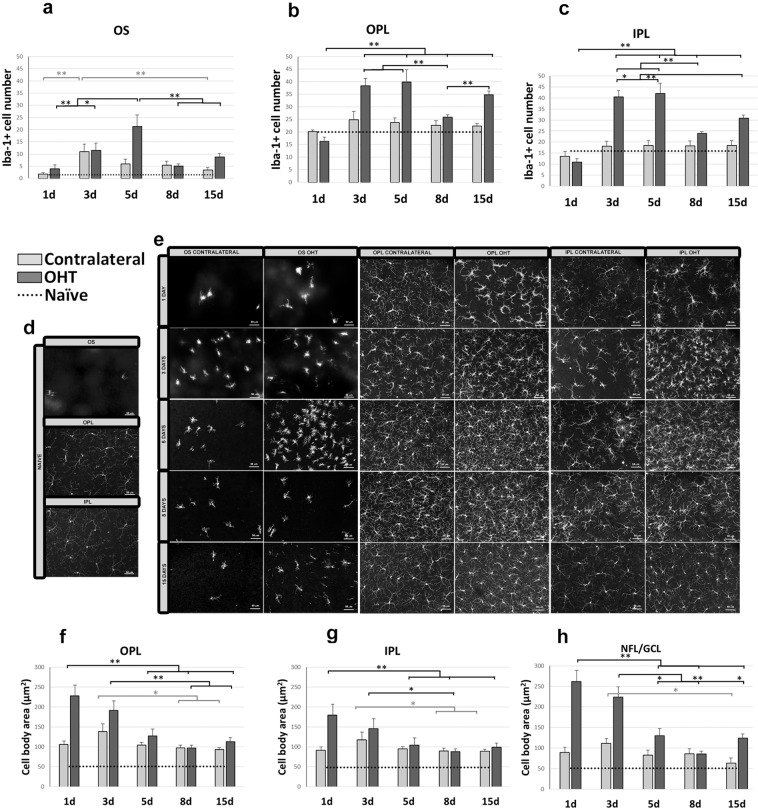
Iba-1+ cell number in the photoreceptor outer segment layer (OS), outer plexiform layer (OPL) and inner plexiform layer (IPL) and cell body area of Iba-1+ cells in the OPL, IPL and the nerve fiber-ganglion cell layer (NFL-GCL) at different times after laser-induced ocular hypertension (OHT). (**a–c**) Variation over time in the number of Iba-1+ cells. Histograms compare the mean (±SD) number of Iba-1+ cells per area of 0.1502 mm^2^ at 1, 3, 5, 8 and 15 d between eyes with ocular hypertension (OHT) and contralateral eyes in OS (**a**), OPL (**b**) and IPL (**c**). (**d,e**) Immunohistochemical images of anti-Iba-1-stained retinal whole-mounts (**d:** naïve; **e:** OHT and contralateral). **OS (a,e)** In OHT eyes, the number of Iba-1+ cells increased from 1 to 5 d, then decreased at 8 and 15 d. In contralateral eyes, the number of Iba-1+ cells increased from 1 to 3 d, then decreased slightly until 15 d. **OPL** and **IPL (b,c,e)** In OHT eyes, the largest increase in the number of microglial cells from 1 d occurred at 3 and 5 d. The number decreased from 5 d until 8 d and 15 d. (**f–h**) Variation over time in the cell body area of Iba-1+ cells after laser-induced OHT. Histograms show mean (±SD) cell body area of Iba-1+ cells in OHT eyes and contralateral eyes at 1, 3, 5, 8 and 15 d in OPL (**f**), IPL (**g**) and NFL-GCL (**h**). **OPL-IPL** (**e**,**f**,**g**) The cell body area in OHT eyes was significantly larger at 1 d, then significantly decrease at 5, 8 or 15 d. In contralateral eyes, the cell body area was greatest at 3 d, and then it decreased significantly at 8 and 15 d. **NFL/GCL** (**h**) In OHT eyes, the cell body area was largest at 1 d, and then it progressively decreased at 5, 8 and 15 d. In contralateral eyes, the cell body area was greater at 3 d, and then it decreased such that it was significantly smaller at 15 d than at 3 d. The dotted line indicates the mean values in naïve eyes. *p < 0.05, **p < 0.01.

The number of Iba-1+ cells in the outer segment layer (OS) was significantly higher in OHT eyes than in naïve eyes at 3, 5, 8, and 15 d (all p < 0.01; Supplementary Fig. S1b–e; Fig. 2a,d,e); similarly, the number was significantly higher in contralateral eyes than in naïve eyes at 3, 5, and 8 d (all p < 0.01; Supplementary Fig. S1b–e; Fig. 2a,d,e). The variation in OS of OHT and contralateral eyes over time showed that the number of Iba-1+ cells in OHT eyes was significantly higher at 5 d than at 1 d (p < 0.01) or 3 d (p < 0.05), while the numbers at 8 and 15 d were significantly lower than the peak at 5 d (both p < 0.01; Fig. 2a,e). The peak number of Iba-1+ cells in contralateral eyes occurred at 3 d (p < 0.01 vs 1 day; Fig. 2a,e), after which the number decreased significantly by 15 d (p < 0.01 vs 3 d), becoming similar to the number in naïve eyes (Fig. 2a,d,e).

In OHT eyes, no differences were found in the numbers of Iba-1+ cells among the superior, inferior, nasal and temporal retinal zones, either when the analysis was performed over all retinal areas or over the areas nearest to the optic disc.

### Changes in Iba-1**+** cells in the plexiform layers

#### Comparison of OHT and contralateral eyes with naïve eyes (OPL and IPL)

OHT and contralateral eyes showed several significant differences from naïve eyes in the outer plexiform layer (OPL) and inner plexiform layer (IPL). The number of Iba-1+ cells was significantly higher in OHT eyes than in naïve eyes at 3, 5, and 15 d in both OPL and IPL (all p < 0.01; Supplementary Fig. S1b,c,e; Fig. 2b–e) and at 8 d in IPL (p < 0.01; Supplementary Fig. S1d; Fig. 2c–e). The number was significantly higher in contralateral eyes than in naïve eyes only at 8 d in IPL (p < 0.05; Supplementary Fig. S1d; Fig. 2c–e). The number of Iba-1+ cells in the OPL was significantly higher in OHT eyes than in contralateral eyes at 3 d (p < 0.05; Supplementary Fig. S1b; Fig. 2b,d,e), 5 d (p < 0.05; Supplementary Fig. S1c; Fig. 2b,d,e) and 15 d (p < 0.01; Supplementary Fig. S1e; Fig. 2b,d,e). A similar result was observed in the IPL at 3, 5 and 8 d (all p < 0.05; Supplementary Fig. S1b,c,d; Fig. 2c–e) as well as 15 d (p < 0.01; Supplementary Fig. S1e; Fig. 2c–e). Soma size was significantly greater in OHT and contralateral eyes than in naïve eyes at all time points (all p < 0.01; Supplementary Fig. S1f–j; Fig. 2d–g).

In OHT eyes, no differences were found in the numbers of Iba-1+ cells among the superior, inferior, nasal and temporal retinal zones in the OPL or IPL, either when the analysis was performed over all retinal areas or over the areas nearest to the optic disc.

**Figure 3:**
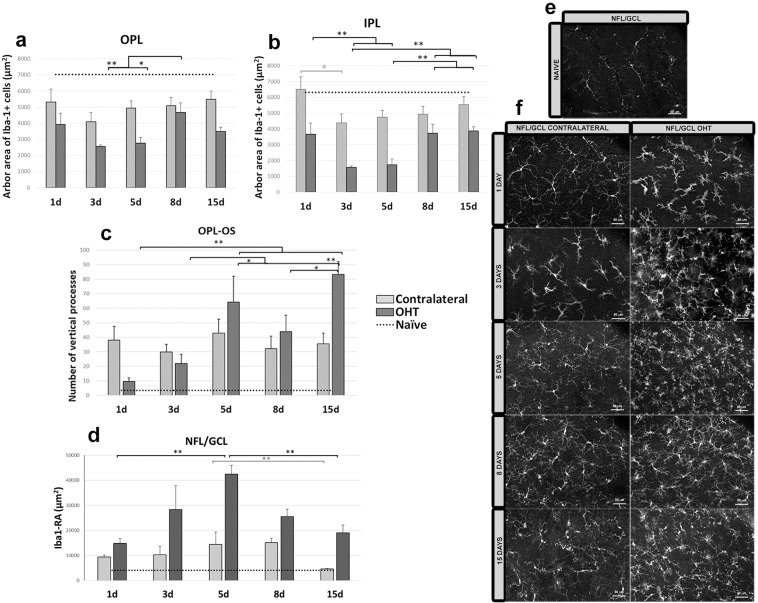
Arbor area (AA) of Iba-1 + cells in the outer plexiform layer (OPL) and inner plexiform layer (IPL); Iba-1 + vertical processes (VP) between OPL and the photoreceptor outer segment layer (OS) and retinal area occupied by Iba-1+ cells (Iba1-RA) in the nerve fiber-ganglion cell layer (NFL-GCL) at different times after laser-induced ocular hypertension (OHT). (**a,b**) Variation over time in AA. Histograms show a mean (±SD) AA in OHT eyes and contralateral eyes at 1, 3, 5, 8 and 15 d in OPL (**a**) and IPL (**b**). In OHT eyes, AA in the OPL decreased marginally from 1 d to 3 d, and then it increased significantly at 8 d. AA in the IPL decreased significantly from 1 d to 5 d. Then the area increased from 5 d to 15 d. In contralateral eyes, AA in the OPL and IPL was smaller at 3 d than at 1 d, and it was significantly noticeable only in the IPL. From 3 d onward, the AA in OPL and IPL did not change significantly. (**c**) Variation over time in the number of microglial VP. The histogram shows a mean (±SD) number of microglial VP between OPL and OS in OHT eyes and contralateral eyes at 1, 3, 5, 8 and 15 d. In OHT eyes, the number of VP increased progressively from 1 to 15 d. In contrast, the number of vertical processes in contralateral eyes did not change significantly between 1 and 15 d. (**d**) Variation in the Iba1-RA over time after laser-induced OHT. The histogram shows a mean (±SD) Iba1-RA in OHT eyes and contralateral eyes at 1, 3, 5, 8 and 15 d. Iba1-RA was significantly greater at 5 d than at 1 d in OHT eyes (significantly) and in contralateral eyes (marginally), after which it decreased such that it was significantly lower at 15 d than at 5 d in both eyes. (**e,f**) Immunohistochemical images of anti-Iba-1 retinal whole-mounts (**e)** naïve; (**f**) OHT and contralateral). The dotted line indicates the mean values in naïve eyes. *p < 0.05, **p < 0.01.

At all time points, the arbor area of Iba-1+ cells in the OPL was significantly smaller in OHT eyes than in naïve eyes at 1 d (p < 0.05; Supplementary Fig. S2a; Fig. 3a,e,f) as well as at 3, 5, 8 and 15 d (all p < 0.01; Supplementary Fig. S2b–e; Fig. 3a,e,f), indicating a retraction of processes. Similarly, the area in IPL was significantly smaller in OHT eyes than in naïve eyes at 1, 3, 5, 8 and 15 d (all p < 0.01; Supplementary Fig. S2a–e; Fig. 3b,e,f). These results with OHT eyes were mirrored in those with contralateral eyes, in which the arbor area in OPL was significantly smaller than in naïve eyes at 3, 5 and 8 d (all p < 0.01; Supplementary Fig. S2b–d; Fig. 2a,e,f) as well as 15 d (p < 0.05; Supplementary Fig. S2e; Fig. 3a,e,f). The arbor area in IPL was significantly smaller in contralateral eyes than in naïve eyes at 3, 5, 8 and 15 d (all p < 0.01; Supplementary Fig. S2b–e; Fig. 3b,e,f). Arbor areas in the OPL were significantly larger in contralateral than OHT eyes at 3 and 5 d (both p < 0.05; Supplementary Fig. S2b–c; Fig. 3a,e,f); areas in the IPL were significantly larger in contralateral than OHT eyes at 1, 3, 5 and 8 d (all p < 0.05; Supplementary Fig. S2a–d; Fig. 3b,e,f) as well as 15 d (p < 0.01; Supplementary Fig. S2e; Fig. 3b,e,f).

The number of vertical processes between the OPL and OS was significantly greater in OHT eyes than in naïve eyes at all time points (p < 0.01; Supplementary Fig. S2g–j; Fig. 3c) except at 1 d (Supplementary Fig. S2f, Fig. 3c); the number of processes was significantly greater in contralateral eyes than in naïve eyes at all time points (p < 0.01; Supplementary Fig. S2f–j; Fig. 3c). At 1 d, the number of processes was significantly greater in contralateral than OHT eyes (p < 0.05; Supplementary Fig. S2f; Fig. 3c). However, at 15 d, the number of processes in OHT eyes was significantly greater than in contralateral eyes (p < 0.01; Supplementary Fig. S2j; Fig. 3c).

#### Variation in plexiform layers of OHT and contralateral eyes over time

*Number of Iba-1*+ *cells in OPL and IPL*. In OHT eyes, the largest increase in the number of Iba-1+ cells from 1 d occurred at 3 and 5 d (both p < 0.01; Fig. 2b,c,e). The number decreased from 5 d until 8 d and 15 d (both p < 0.01; Fig. 2b,c,e).

*Iba-1*+ *cell body area in OPL and IPL*. Soma size in OHT eyes was significantly greater at 1 d than at 5, 8 or 15 d (all p < 0.01; Fig. 2f,g). Soma size in the OPL decreased marginally from 1 to 3 d, after which it decreased significantly, such that it was significantly smaller at 8 and 15 d than at 3 d (both p < 0.01; Fig. 2f). Similar results were observed in the IPL, where size was significantly smaller at 8 d than at 3 d (p < 0.05; Fig. 2g). In contrast, soma size in contralateral eyes was greatest at 3 d, after which it decreased such that it was significantly smaller at 8 and 15 d than at 3 d (both p < 0.05; Fig. 2f,g).

*Arbor area of the Iba-1*+ *cells in the OPL and IPL*. In OHT eyes, Iba-1+ arbor area in the OPL decreased marginally from 1 d to 3 d, after which it increased, such that the area was significantly greater at 8 d than at 3 d (p < 0.01; Fig. 3a) and greater at 8 d than at 5 d (p < 0.05; Fig. 3a). Arbor area in the IPL decreased significantly from 1 d to 5 d, such that the area at 3 and 5 d was significantly smaller than at 1 d (both p < 0.01; Fig. 3b). Area increased from 5 d, such that area at 8 and 15 d was significantly greater than at 3 or 5 d (all p < 0.01; Fig. 3b).

In contralateral eyes, arbor area in the OPL and IPL was smaller at 3 d than at 1 d, although this decrease was significant only in the IPL (p < 0.05; Fig. 3b). From 3 d onward, arbor area in OPL and IPL did not change significantly (Fig. 3a,b).

*Iba-1*+ *vertical processes between OPL and OS*. In OHT eyes, the number of vertical processes increased progressively from 1 to 15 d: the numbers at 5 and 15 d were greater than at 1 d (both p < 0.01; Fig. 3c), the numbers at 5 and 15 d were greater than at 3 d (p < 0.05 and p < 0.01, respectively; Fig. 3c), and the number at 15 d was greater than at 8 d (p < 0.05; Fig. 3c). In contrast, the number of vertical processes in contralateral eyes did not change significantly between 1 and 15 d (Fig. 3c).

### Changes in the Iba-1+ cell in the nerve fiber layer-ganglion cell layer (NFL-GCL)

The changes suffered in Iba-1+ cells of this layer after laser-induced OHT made it difficult to quantify their number. We quantified the area of the retina occupied by Iba-1+ cells (abbreviated Iba1-RA) as in previous studies^9,32,33^ and we determined Iba-1+ soma area.

#### Comparison of OHT and contralateral eyes with naïve eyes

OHT and contralateral eyes showed several significant differences from naïve eyes. Iba1-RA was significantly greater in OHT eyes than in naïve eyes at all time points (p < 0.01; Supplementary Fig. S3a–e; Fig. 3d–f), and it was significantly greater in contralateral eyes than in naïve eyes at 1,3 and 5 d (in all cases p < 0.01; Supplementary Fig. S3a–c; Fig. 3d–f) and at 8 and 15 d (both p < 0.05; Supplementary Fig. S3d,e; Fig. 3d–f). In addition, Iba1-RA was significantly greater in OHT than in contralateral eyes at 1, 3, 5 and 8 d (p < 0.05; Supplementary Fig. S3a–d; Fig. 3d,f) as well as 15 d (p < 0.01; Supplementary Fig. S3e; Fig. 3d,f).

In OHT eyes, no differences were found in Iba1-RA among the superior, inferior, nasal and temporal retinal zones, either when the analysis was performed over all retinal areas or over the areas nearest to the optic disc.

Soma size was significantly greater in OHT eyes than in naïve eyes at all time points (all p < 0.01; Supplementary Fig. S1f–j; Fig. 2h); it was greater in contralateral eyes than naïve eyes at 1, 3, 5, and 8 d (all p < 0.01; Supplementary Fig. S1f–I; Fig. 2h) as well as 15 d (p < 0.05; Supplementary Fig. S1j; Fig. 2h) Soma size was significantly greater in OHT than in contralateral eyes at 1, 3, 5 and 15 d (all p < 0.05; Supplementary Fig. S1f,g,h,J; Fig. 2h).

#### Variation in OHT and contralateral eyes over time (NFL-GCL)

In OHT eyes, Iba1-RA was significantly greater at 5 d than at 1 d (p < 0.01; Fig. 3d,f), after which it decreased such that it was significantly lower at 15 d than at 5 d (p < 0.01; Fig. 3d,f). In contralateral eyes, Iba1-RA was marginally larger at 5 d than at 1 d, after which it decreased such that it was significantly smaller at 15 d than at 5 d (p < 0.05; Fig. 3d,f).

In OHT eyes, the cell body area was largest at 1 d, and it decreased progressively such that it was significantly smaller at 5, 8 and 15 d than at 1 d (all p < 0.01; Fig. 2h). Size was significantly smaller at 5 and 15 d than at 3 d (both p < 0.05; Fig. 2h), and it was significantly smaller at 8 d than at 3 d (p < 0.01; Fig. 2h). In contralateral eyes, in contrast, soma size was greater at 3 d, and then it decreased such that it was significantly smaller at 15 d than at 3 d (p < 0.05; Fig. 2h).

#### P2RY12 expression over time

In order to determine whether most Iba1+ cells were activated microglia or infiltrating macrophages in our experiments, we performed double immunostaining against Iba-1 and P2RY12.

**Figure 4:**
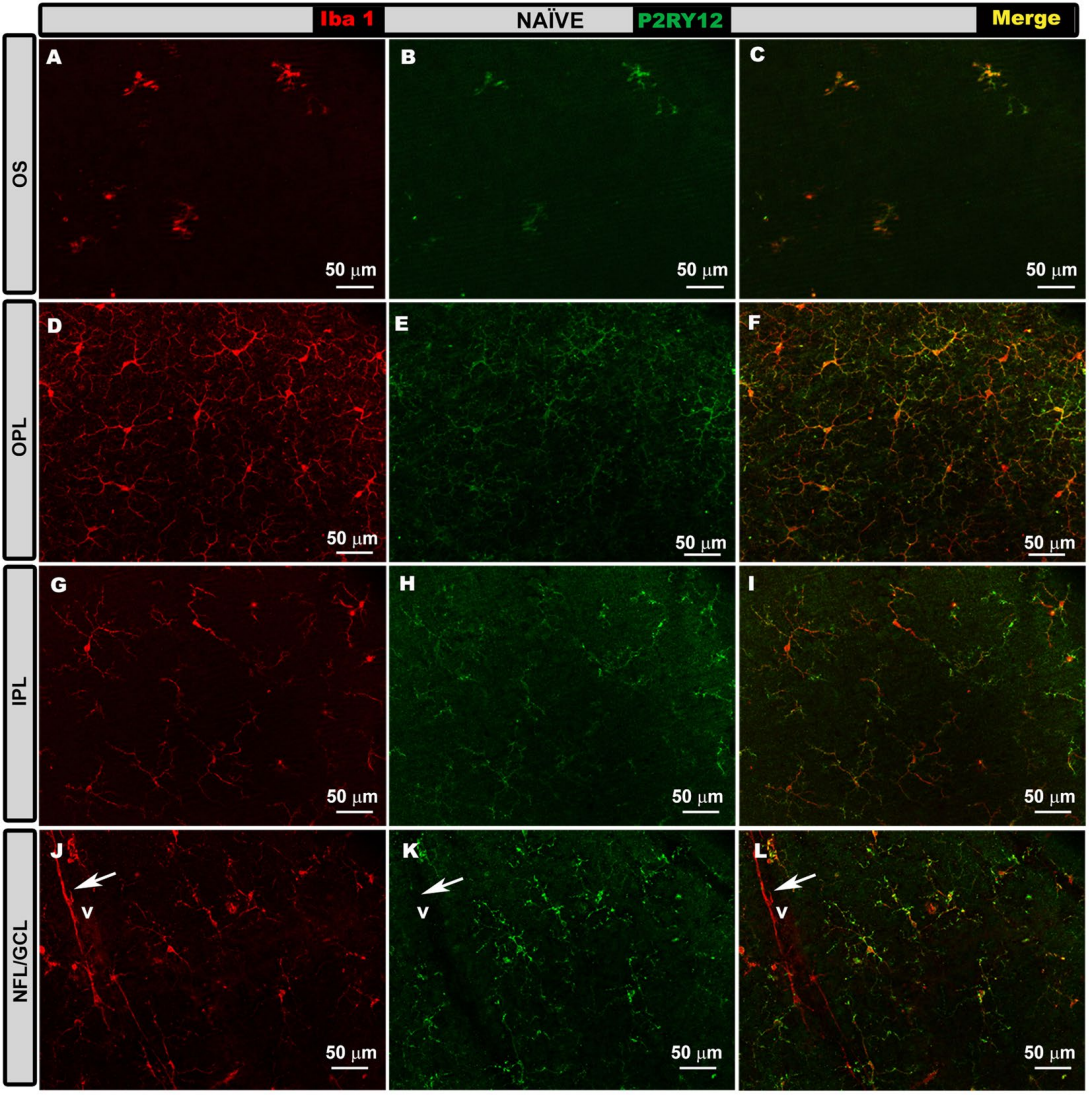
P2RY12 expression in naïve eyes in the photoreceptor outer segment layer (OS), outer plexiform layer (OPL) inner plexiform layer (IPL) and in the nerve fiber-ganglion cell layer (NFL-GCL). Double immunostaining: Iba-1 (**A**,**D**,**G**,**J**), P2RY12 (**B**,**E**,**H**,**K**), merge (**C**,**F**,**I**,**L**). Retinal whole-mounts. In all retinal layers analyzed, Iba-1+ cells showed P2RY12 expression, except perivascular iba-1+ cells (**j–l**), which were P2RY12- (arrow). V: vessel.

Naïve eyes. In all retinal layers, Iba-1+ cells expressed P2RY12 (Fig. 4a–l), with the exception of perivascular microglia (Fig. 4j–l), which were located in some retinal vessels in the NFL, and dendritic-like Iba-1+ cells located in the juxtapapillary area and peripheral retina in the IPL. The mean values of P2RY12 expression intensity in naïve eyes were (OPL = 18.47 ± 3.16; IPL = 32.21 ± 4.44 and NFL-GCL = 14.35 ± 4.28) (Fig. 4).

**Figure 5:**
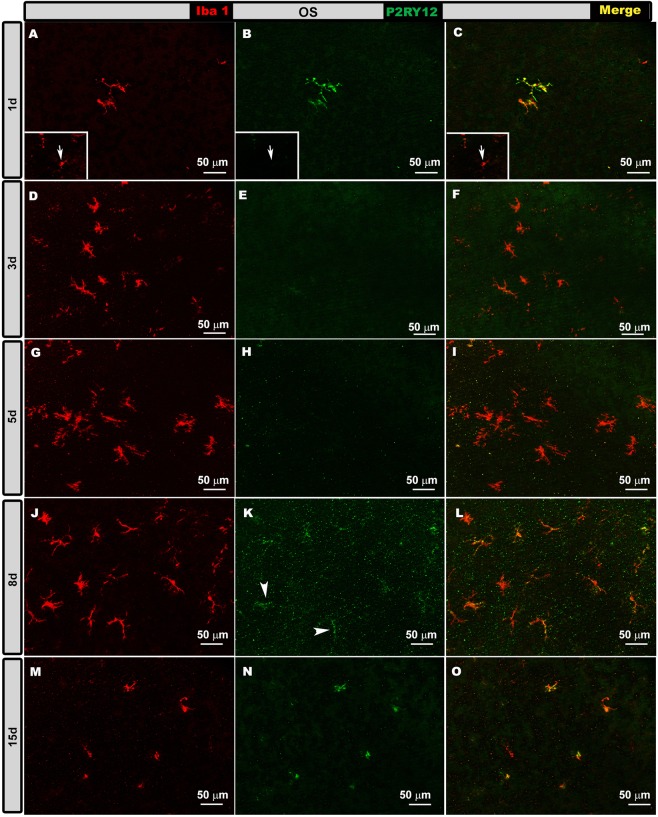
P2RY12 expression in ocular hypertension (OHT) eyes in the photoreceptor outer segment layer (OS) at different times after laser-induced OHT (1, 3, 5, 8 and 15 d). Double immunostaining: Iba-1 (**A,D,G,J,M**), P2RY12 (**B,E,H,K,N**), merge (**C,F,I,L,O**). Retinal whole-mounts. At 1 and 15 d after laser induction, most Iba-1+ cells showed strong P2RY12 expression in OS. At 3 and 5 d, P2RY12 expression in Iba-1+ cells was substantially down-regulated in this layer; it increased slightly at 8 d (arrowheads), reaching values similar to those of naïve eyes at 15 d. Rounded cells were P2RY12- (arrow) (inset **A–C**).

**Figure 6:**
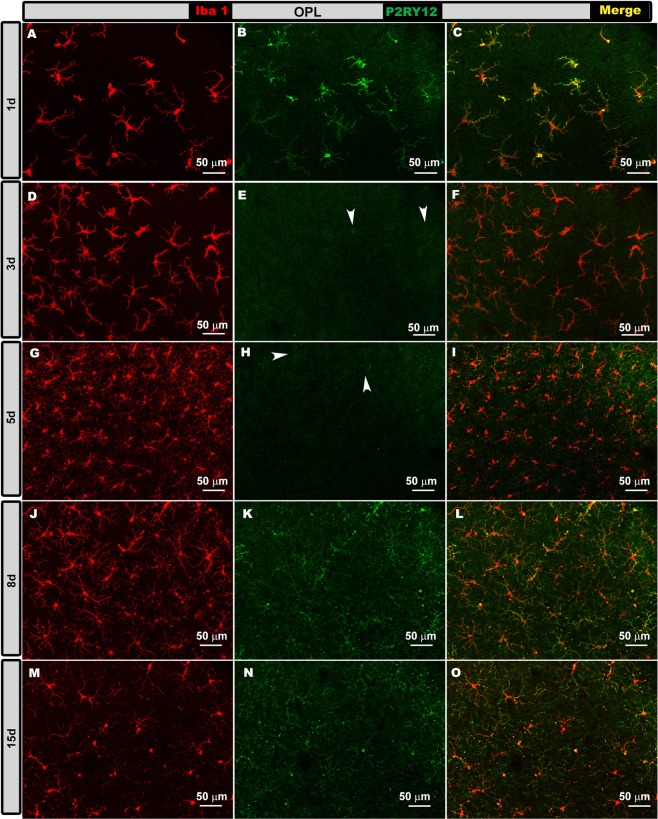
P2RY12 expression in ocular hypertension (OHT) eyes in the outer plexiform layer (OPL) at different times after laser-induced OHT (1, 3, 5, 8 and 15 d). Double immunostaining: Iba-1 (A,D,G,J,M), P2RY12 (B,E,H,K,N), merge (**C,F,I,L,O**). Retinal whole-mounts. At 1 d after laser induction, most Iba-1+ cells intensely expressed P2RY12. This expression was strongly reduced at 3 and 5 d (arrowheads); it increased weakly at 8 d, reaching values similar to those of naïve eyes at 15 d.

**Figure 7:**
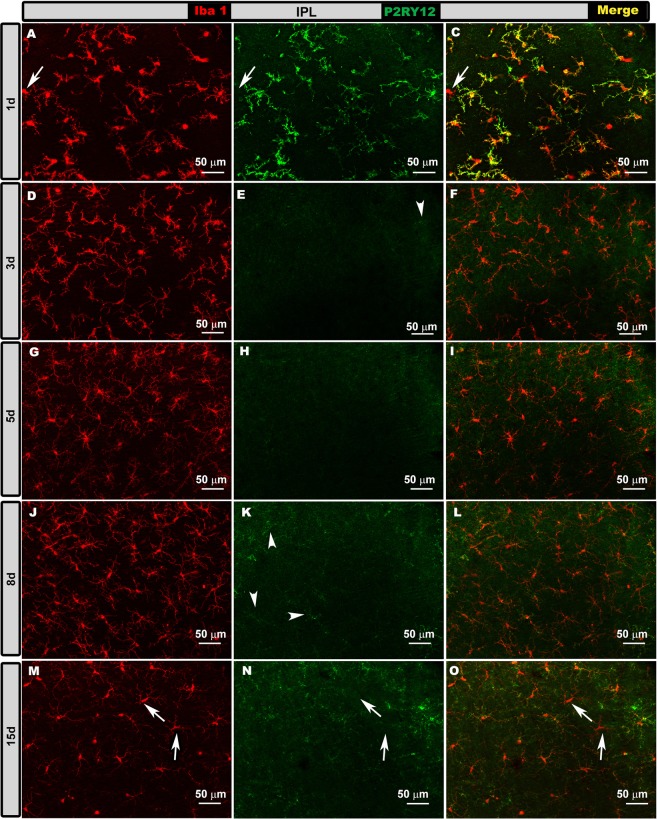
P2RY12 expression in ocular hypertension (OHT) eyes in the inner plexiform layer (IPL) at different times after laser-induced OHT (1, 3, 5, 8 and 15 d). Double immunostaining: Iba-1 (**A,D,G,J,M**), P2RY12 (**B,E,H,K,N**), merge (**C,F,I,L,O**). Retinal whole-mounts. At 1 d after laser induction, most Iba-1+ cells strongly expressed P2RY12, but some amoeboid cells did not express this marker (arrow in **A–C**). At intermediate times (3 and 5 d), P2RY12 expression was much lower. At 8 d, this expression increased weakly (arrowheads) to reach values similar to those in naïve eyes at 15 d. At 15 d, a few ramified Iba-1+ cells were P2RY12- (arrows).

**Figure 8:**
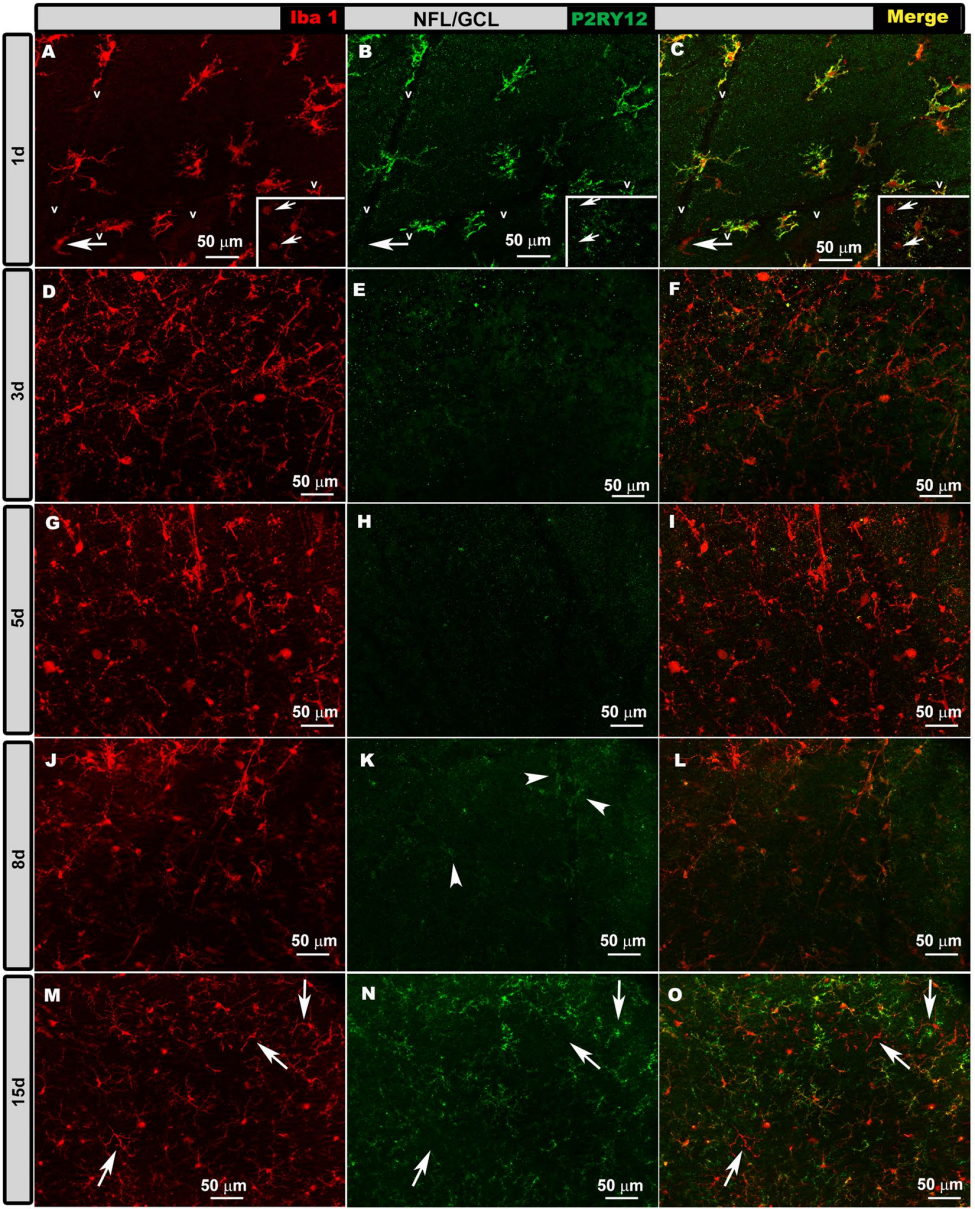
P2RY12 expression in ocular hypertension (OHT) eyes in the nerve fiber-ganglion cell layer (NFL-GCL) at different times after laser-induced OHT (1, 3, 5, 8 and 15 d). Double immunostaining: Iba-1 (**A,D,G,J,M**), P2RY12 (**B,E,H,K,N**), merge (**C,F,I,L,O**). Retinal whole-mounts. At 1 d, most Iba-1+ cells stained intensely as P2RY12+, while only a few rounded cells (inset A-C arrows) and perivascular cells (arrow) were P2RY12-. At 3 and 5 d, P2RY12 expression in Iba-1 + cells was dramatically reduced in this layer, then it increased slightly at 8 d (arrowheads) to reach values similar to those in naïve eyes at 15 d. At 15 d, a few ramified Iba-1+ cells did not express P2RY12 (arrows).

OHT eyes. At 1d and 15 d after laser induction, most Iba-1+ cells showed high P2RY12 expression in the OS, OPL, IPL and NFL/GCL (Figs. 5–8, panels a-c, m-o in all figures), except in the following cases: i) perivascular and dendritic-like cells, ii) some rounded cells in the OS (Fig. 5a–c, inset) and NFL/GCL (Fig. 8a–c, inset), and iii) very few ramified cells in the IPL (Fig. 7m–o) and NFL/GCL (Fig. 8m–o). At intermediate times (3 and 5 d), P2RY12 expression in Iba-1+ cells was dramatically lower than at 24 h in all retinal layers (Figs. 5–8, panels d-i in all figures). Expression increased weakly at 8 d (Figs. 5–8, panels j-l in all figures), reaching values similar to those in naïve eyes at 15 d (Figs. 5–8, panels m-o in all figures).

The mean values of P2RY12 expression intensity in OHT eyes were as follows at 1d (OPL = 25.62 ± 3.20, p < 0.01 vs Naïve; IPL = 33.63 ± 2.40 and NFL-GCL = 12.63 ± 0.92); 3d (OPL = 5.47 ± 0.46; IPL = 1.33 ± 0.06 and NFL-GCL = 2.99 ± 0.38; all p < 0.01 vs Naïve); 5d (OPL = 5.95 ± 0.76; IPL = 2.78 ± 0.34 and NFL-GCL = 1.28 ± 0.10; all p < 0.01 vs Naïve); 8d (OPL = 16.65 ± 3.98; IPL = 22.30 ± 2.76, p < 0.05 vs Naïve; and NFL-GCL = 6.80 ± 2.34, p < 0.01 vs Naïve) and 15d (OPL = 18.81 ± 5.28; IPL = 33.91 ± 6.14 and NFL-GCL = 13.44 ± 5.22). (Fig. 4).

**Figure 9:**
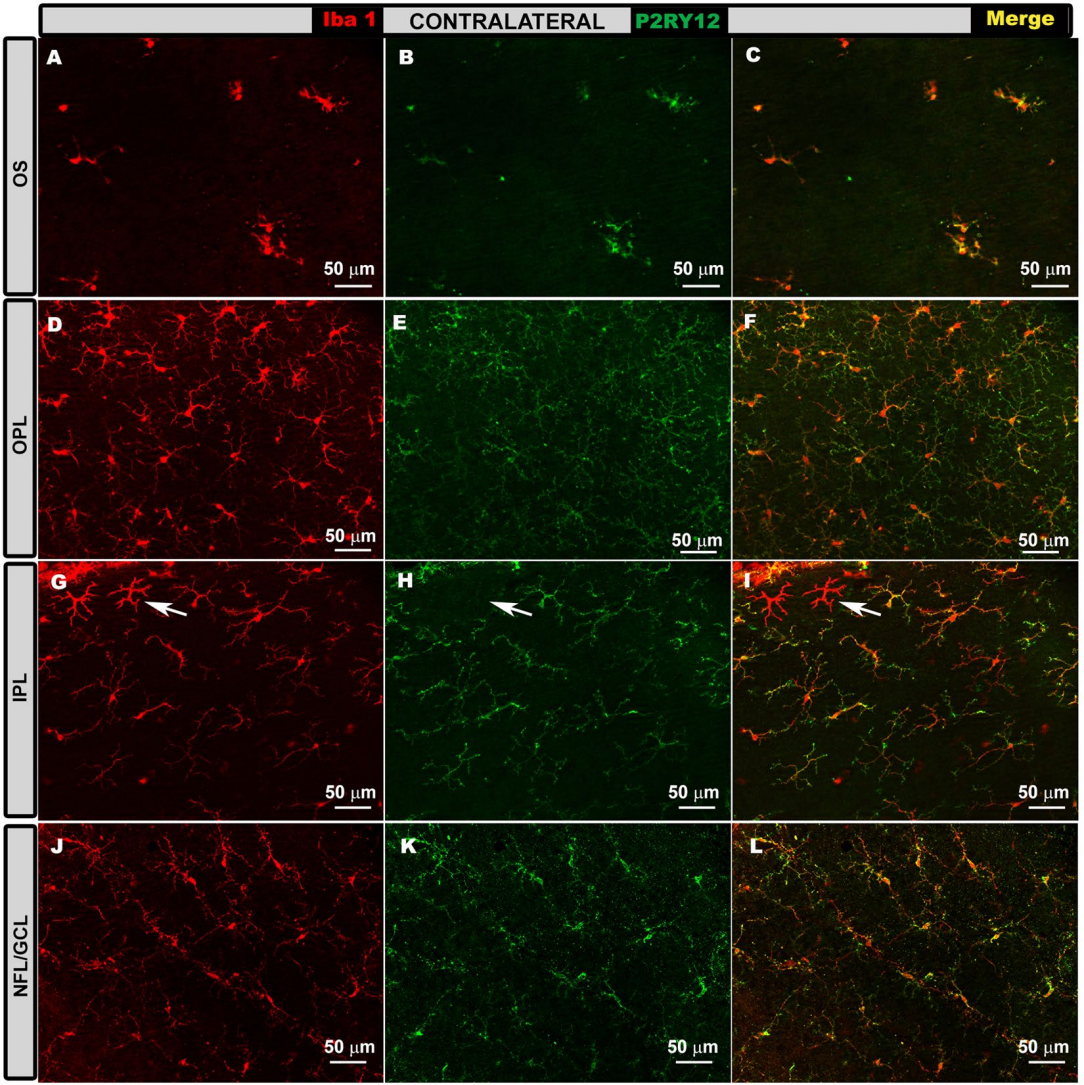
P2RY12 expression in contralateral eyes in the photoreceptor outer segment layer (OS), outer plexiform layer (OPL) inner plexiform layer (IPL) and in the nerve fiber-ganglion cell layer (NFL-GCL). Double immunostaining: Iba-1 (**A,D,G,J**), P2RY12 (**B,E,H,K**), merge (**C,F,I,L**). Retinal whole-mounts. In all retinal layers analyzed, most Iba-1+ cells were labelled with anti-P2RY12 antibody at all time points analyzed: 1 d (**A–C**), 3 d (**G–H**), 5 d, 8 d and 15 d (**D–F**,**J–L**). P2RY12 expression was similar in all instances. This was not the case for dendritic-like cells (arrow in G-I) or perivascular cells, which were P2RY12-.

Contralateral eyes. In contralateral eyes, as in naïve ones (Fig. 4), Iba-1+ cells were intensely labelled with anti-P2RY12 antibody (Fig. 9), while dendritic-like cells (Fig. 9g–i) and perivascular cells did not show staining. P2RY12 expression was similar in all retinal layers at all time points (24 h, 3 d, 5 d, 8 d and 15 d; Fig. 9). The mean values of P2RY12 expression intensity in contralateral eyes were as follows at 1d (OPL = 23.21 ± 6.14; IPL = 28.03 ± 7.86 and NFL-GCL = 12.75 ± 3.92); 3d (OPL = 25.45 ± 2.84, p < 0.05 vs Naïve; p < 0.05 vs OHT; IPL = 37.64 ± 4.12, p < 0.05 vs Naïve; p < 0.05 vs OHT; and NFL-GCL = 13.96 ± 1.76, p < 0.05 vs OHT); 5d (OPL = 26.35 ± 4.64, p < 0.05 vs Naïve; p < 0.05 vs OHT; IPL = 38.40 ± 1.80, p < 0.05 vs Naïve; p < 0.05 vs OHT, and NFL-GCL = 12.97 ± 2.16, p < 0.05 vs OHT); 8d (OPL = 23.35 ± 4.78, p < 0.05 vs Naïve; IPL = 31.13 ± 4.22 and NFL-GCL = 14.51 ± 4.64, p < 0.05 vs OHT); and 15d (OPL = 19.22 ± 4.40; IPL = 34.86 ± 7.86 and NFL-GCL = 11.69 ± 5.00) (Fig. 4).

## Discussion

This work is, to the best of our knowledge, the first comparative study of microglial activation among different time points (1, 3, 5, 8 and 15 days) after unilateral laser induction of OHT in an experimental mouse model. It is also the first study of this OHT mouse model in which P2RY12 expression is analyzed over time in retinal whole-mounts. Microglial activation, measured here in terms of morphology and number of Iba-1+ cells, was observed in all retinal layers where these cells are present, i.e. the OS, OPL, IPL, and GCL-NFL. Most studies of microglial activation over time after OHT have involved a DBA/2 J mouse model of inherited glaucoma^34–38^. Other studies have relied on rat glaucoma models, in which OHT is induced differently than in our model^21,39–45^.

In the healthy retina, microglial cells remain in a quiescent state characterized by a ramified morphology. In the presence of damage, such as OHT, they become activated, their processes shorten, and their soma enlarges. In their state of high activation, they take on amoeboid morphology and behavior, acting as macrophages to engulf debris^9,16,32,38,46,47^. The abundance of microglia increases upon activation as a result of migration and/or proliferation^9,32,36,40,48–52^. All these signs of microglial activation were observed in the present study, in OHT eyes and the contralateral normotensive eyes. We are confident that microglial activation was triggered by the increase in IOP and not by an inflammatory response to laser treatment because, in our previous work with the same OHT model^48^, no morphological signs of microglial activation were detected in animals exposed to laser in the non-draining portion of the sclera, and these eyes showed similar IOP as naïve ones.

Laser induction of OHT in the present study considerably increased IOP at 1 d later. This increase remained at 3 d and then gradually returned to the same pressure as in contralateral eyes at 8 d. They were consistent with several previous studies using the same model^53,54^.

To label microglial cells, we used anti-Iba-1 antibody, which can reveal morphological features of activated microglial cells^55^. This antibody is expressed in the cells of monocytic lineages, including microglia, dendritic cells, monocytes and macrophages. Thus, it cannot distinguish activated microglia from infiltrating macrophages. In order to determine whether Iba-1+ cells in our study were mainly microglial cells, we stained tissue with antibody against P2RY12, which is expressed by microglia but not by monocytes, infiltrating macrophages or dendritic cells^27,56^.

Resting microglia express abundant P2RY12, which is up-regulated after injury by ATP released from damaged cells. The P2RY12 triggers the extension of microglial process towards lesions. A few hours later, P2RY12 expression is down-regulated^22^. In fact, down-regulation of P2RY12 mRNA and protein levels is one of the most sensitive markers for the transition from resting to activated microglia^26^. In our study, in naïve retinas, all Iba-1+ cells expressed P2RY12, with the exception of perivascular and dendritic-like cells. At 24 h after OHT induction, most Iba-1+ cells in the OS, plexiform layers and NFL-GCL showed abundant P2RY12 expression. This expression was down-regulated at 3 and 5 d, then it rose weakly at 8 d, reaching values similar to those of naïve eyes at 15 d. Consistent with previous studies, our results suggest that the P2RY12 receptor is down-regulated at 3 and 5 d, when the inflammation in our model was strongest.

As expected, neither perivascular nor dendritic Iba-1+ cells showed P2RY12 inmunostaining in our study. Similarly, the sparse rounded cells observed at 1 and 15 d in the NFL-GCL and OS did not show P2RY12 expression, so they may be macrophages or monocytes infiltrating the bloodstream. P2RY12 is selectively expressed in resident microglia, and its expression correlates strongly with a ramified microglial morphology^29^. Most Iba-1+ cells with a ramified and macrophagic appearance were P2RY12+. The few ramified and macrophagic P2RY12- cells were located primarily in the IPL and NFL-GCL and may derive from bloodstream-infiltrating macrophages.

When we analyzed the morphological signs of activation, we found that in the OS, the number of Iba-1+ cells was much higher in OHT and contralateral eyes than in naïve eyes at all time points after 1 d. In OHT eyes, the number increased progressively from 1 until 5 d, when it peaked, after which the number decreased. The increase in cell number in the OS may be related to the increase in IOP. Microglial activation promotes a pro-inflammatory environment that can affect retinal pigment epithelium morphology and function^57,58^. Activated Iba-1+ cells in the OS produce pro-inflammatory factors and chemokines that can trigger rupture of the external blood-retina barrier, inducing blood-cell recruitment^59^. In a study using the same OHT model as the present study, cells possibly coming from the bloodstream in the OS were observed at 15 d^32^.

In the plexiform layers, somata of Iba-1+ cells were significantly larger and arbor area significantly smaller in contralateral and OHT eyes than in naïve eyes at all time points after OHT induction. These effects were more pronounced in OHT than in contralateral eyes. In OHT, somata of Iba-1+ cells began to enlarge and processes to shorten beyond 1 d; retraction was maximum at 3 and 5 d, and somata were still large, although smaller than at 1 d. The number of Iba-1+ cells peaked at these times. From these times through the end of the 15 d, soma area decreased slightly, arbor area increased and the number of Iba-1+ cells fell, though it remained higher than in naïve eyes. Therefore, microglial activation in the plexiform layers of hypertensive eyes was greater at 3 and 5 d after OHT induction, when IOP was highest (1, 3 and 5 d).

OHT-induced microglial activation may be driven in part by the expression of growth factors, some of which are secreted by activated macroglia^52^. In a previous study, using the same experimental model as the present work, we observed activation of astrocytes and Müller glia^9^, which may have contributed to the increase in number of Iba-1+ cells in plexiform layers in OHT eyes in the current work. Previous studies using rodent models of laser-induced OHT have shown functional alterations in full-field electroretinogram recordings^60–62^. Indeed, the amplitude of the positive scotopic threshold response associated with retinal ganglion cell activity was significantly lower in OHT eyes than in naïve eyes throughout the 56-d measurement period. This difference was associated with a significant reduction in the a- and b-waves of the electroretinogram^60,61^. Moreover, substantial morphological alterations of the outer and inner retinal layers have been observed in the laser-induced OHT model, when rodents were analyzed for longer periods^61,62^. Thus, it is possible that neuronal damage in these layers may help explain the microglial activation observed in our study in the plexiform layers and OS.

In our study, soma area and Iba1-RA were higher in OHT and contralateral eyes than in naïve eyes in the NFL-GCL at all time points analyzed. These effects were more pronounced in OHT than contralateral eyes. In OHT eyes, Iba1-RA increased progressively from 1 d until its peak at 5 d, after which it decreased progressively until 15 d. This increase in Iba1-RA in the NFL-GCL may be explained by the increase in soma area of Iba-1+ cells. It may also be explained by entry of other cell types from the bloodstream, as a result of rupture of the internal blood-retina barrier due to the increased IOP^31,32,41^. A third possible explanation is migration of microglia from the IPL^31^.

Increased IOP triggers retinal ganglion cell degeneration and death^53,60^, which may have contributed to the microglial activation in the NFL-GCL in our study. Damaged neurons release nucleotides that can up-regulate purine receptors in microglia, triggering phagocytosis and migration^63–65^. In addition, deficiencies in the signaling between microglia and retinal ganglion cells activate microglia^66^. Dying neurons release HMGB1, which stimulates the NF-κβ pathway, and NADPH oxidase in microglial cells, thereby inducing the production of multiple inflammatory and neurotoxic factors^67^. A study using the same experimental model as the present work showed a lack of retrograde axonal transport in approximately 75% of the original retinal ganglion cells at 8 d after laser-induced OHT, being focal and diffuse within the retina^53^. In order to ascertain whether the number of Iba-1+ cells depends on sector-specific damage of the retinal ganglion cells, we compared the numbers of Iba-1+ cells by retinal zone (superior, inferior, nasal and temporal) in OHT eyes, over all retinal areas and over areas nearest to the optic disc. No significant sector-specific differences were observed in either case. The generalized increase in the number of Iba-1+ cells across the various retinal zones may be related to the diffuse loss of ganglion cells observed at 8 days after laser induction in this experimental model^60^.

In previous work, we demonstrated microglial activation in the contralateral eye at 1 d^31^ and 15 d^32^ after laser induction of OHT. Here we extend those findings to show that the activation observed at 1 d persisted throughout the full 15 d and that it was independent of IOP elevation, since the pressure was similar in contralateral and naïve eyes. Several findings point to microglial activation in contralateral eyes: (i) more abundant Iba-1+ cells in contralateral than naïve eyes in the OS from 3 d onwards; (ii) larger soma size in contralateral eyes than naïve eyes from 1 d onwards, and smaller arbor area from 3 d in the plexiform layers; (iii) greater Iba1-RA and soma area in contralateral than naïve eyes in the NFL-GCL from 1 d onwards; and (iv) more abundant microglial vertical processes between OPL and OS in contralateral than naïve eyes from 1 d onwards.

Contralateral and naïve eyes showed a similar expression of P2RY12 in all retinal layers and at all time points. These eyes did not show the P2RY12 down-regulation at 3 and 5 d observed in OHT eyes.

Microglial activation in contralateral eyes was less intense than in OHT eyes, and it remained stable over time. In OHT eyes at 1 d, enlargement of somata reached a peak, and processes began to retract. In contralateral eyes, microglia activation was maximal at 3 d when measured in terms of soma area, arbor area, and cell number in OS; or at 5 d when measured in terms of Iba1-RA. These results indicate that microglia in contralateral eyes were activated slightly later than those in OHT eyes. The processes underlying microglial activation in contralateral eyes remain unclear. Such activation does not appear to require neuronal death^9,32,48,60,68^, and it may involve an immune response triggered by impairment of the blood-retina barrier in OHT eyes^31,68^. This may help explain the observed slight delay in microglial activation in contralateral eyes.

In the present studies we have not examined the correlation of microglia activation with RGC loss. However, in a series of previous studies characterizing the model, using the same OHT-induction model and albino Swiss mice it was documented that RGC loss occurred only in the ocular hypertensive eye and not in the contralateral eye^54,60^. In the ocular hypertensive eyes, by 8 days (n = 12) after OHT-induction the population of RGCs retrogradely labelled from their main target in the brain, the superior colliculi, with of OHSt (a small molecule with similar properties to fluorogold), was reduced to approximately 25% of their fellow contralateral population; a loss that did not progressed with further survival intervals of 17 (n = 13), 35 (n = 21), or 63 (n = 13) days)^60^. When the expression of Brn3a was used as a marker to identify surviving RGCs, irrespective or their retrograde axonal transport capabilities, it was shown that by 8 days there was a loss of approximately 50% of the RGC population. Moreover, the study of the spatial distribution of retrogradely labelled RGCs with isodensity maps documented that this loss of RGCs appeared focal in wedge-shaped sectors with their vertex oriented towards the optic nerve head and their base toward retinal periphery as well as diffuse affecting the whole retina^54,60^. Thus, the main loss of RGCs occurs within the first week after OHT-induction and this is the period of time in which iba1+ cells show a peak of activation at 3 and 5d and then activation drops at 8d.

In conclusion, we report the first comparative quantification of diverse signs of microglial activation at different time points after unilateral laser-induced OHT. We examined this activation in OHT and contralateral normotensive eyes. These two eye populations were also compared with naïve eyes to allow rigorous assessment of microglial activation. We observed that microglial cells in OHT and contralateral eyes showed signs of activation at all times after laser induction, and that the number of microglia increased from 3 d onwards. In contralateral eyes microglial activation occurred slightly later, was less intense and more constant than in OHT eyes. Contralateral activation may be trigged by immune signals derived from OHT eyes. Microglial activation was greater in OHT and contralateral eyes at 3 and 5 d, coinciding with high pressure and P2RY12 down-regulation in OHT eyes. These time points of greater inflammation in this glaucoma model may be useful for identifying factors implicated in OHT-induced inflammatory processes.

## Material and Methods

### Animals and anesthetics

In this study adult albino mice (of Swiss strain) of 12-16 weeks of age and 40–45 g of weight were used. The animals, supplied from the breeding colony of the University of Murcia, were maintained in cages with controlled temperature and light (12-h light/dark cycle and 9–24 lux). All mice had ad libitum access to water and food. Induction of ocular hypertension (OHT) and measurement of intraocular pressure (IOP) were done under general anesthesia, using an intraperitoneal (ip) injection of a mixture of xylazine (10 mg/kg; Rompún®, Bayer, Barcelona, Spain) and ketamine (75 mg/kg; Ketolar®, Parke-Davies, Barcelona, Spain). At the time of recovery after anesthesia, a tobramycin ointment (Tobrex®; Alcon, Barcelona, Spain) was applied to the cornea to prevent infection and drying. At all times, attempts were made to reduce pain and discomfort in the animals after surgery. Finally, the animals were sacrificed with an ip overdose of pentobarbital (Dolethal Vetoquinol®, Veterinary Specialties, Alcobendas, Madrid, Spain). All experiments were made in accordance with Spanish law and the Guidelines for Humane Endpoints for Animals Used in Biomedical Research. In addition, the study was accepted by the Ethics Committee for Animal Research of Murcia University (Murcia, Spain) and the Animal Health Service of the Murcia Regional Ministry of Agriculture and Water (approval ID number: A1310110807). Animal procedures were made using the institutional guidelines, European Union regulations for the use of animals in research, and the Association for Research in Vision and Ophthalmology (ARVO) statement for the use of animals in ophthalmic and vision research.

### Experimental groups

Mice were allocated into six groups, each containing 8 animals: one group was composed of naïve age-matched control (naïve) and five experimental groups were treated with laser in the left eye and examined at 1, 3, 5, 8 or 15 d.

### Laser treatment and measurement of IOP

To induce OHT, we treated the left eyes of the mice with a single diode laser session (Viridis Ophthalmic Photocoagulator-532 nm, Quantel Medical, Clermont-Ferrand, France) using the methods described previously^53,61^. Laser beam was applied directly on episcleral and limbal veins, and 55–76 laser burns were performed. The laser parameters were: spot size, 50–100 μm; duration, 0.5 seconds; and power, 0.3 W. IOP reading was performed on the contralateral and laser treated eyes using a rebound tonometer (Tono-Lab, Tiolat, OY, Helsinki, Finland)^40,69^. The intraocular pressure (IOP) was measured in all animals. In the five laser-treated groups, IOP was measured before, and at 1, 3, 5, and 8 d after OHT-induction. In naïve eyes IOP was taken before the animal sacrifice. Six consecutive IOP measurements were taken and then an average was made. IOP was recorded at the same time (approximately 9 a.m.), and just after anesthetizing the animal, to decrease pressure variations due to the animal’s circadian rhythm^70^ or spontaneous increases in the IOP^49^

### Immunohistochemistry

The animals were fixed by transcardiac perfusion. A saline solution was introduced briefly through the aorta and then the fixation solution composed of 4% paraformaldehyde in 0.1 M phosphate buffer (PB, pH 7.4). To maintain the orientation of the eyeball, a stitch was given at the posterior pole. Additional benchmarks such as nasal caruncle and rectus muscles were also used^71^. Subsequently the eyes were post- fixed in 4% paraformaldehyde in 0.1 M phosphate buffer (PB, pH 7.4) for two hours. Then the retinas were separated and the vitreous humor was eliminated to perform retinal whole-mounts^13,72^.

To analyze the microglial population, retina whole-mounts were immunostained as described^9^. Rabbit anti-Iba-1 (Wako, Osaka, Japan) was used as primary antibody after 1:600 dilution in phosphate-buffered saline (PBS) containing 1% donkey serum and 0.1% Triton-X100. The secondary antibody was donkey anti-rabbit antibody conjugated to Alexa Fluor 594 (Invitrogen, Paisley, UK) and diluted 1:800 in PBS.

In order to differentiate the population of retinal activated microglia from infiltrating macrophages, we used an antibody against P2RY12, which reacts with resident microglia, but not with infiltrating monocytes or macrophages^27,73^. For the study of P2RY12 microglial expression, two retinas of each group (naïve, OHT and their contralateral) at the different time points (1, 3, 5, 8 and 15 d, total n = 22) were double-immunostained against Iba-1 and P2RY12. Retinas were pretreated with antigen-unmasking solution (Vector, Burlingame, USA), then incubated with rat anti-P2RY12 (1:100; Biolegend, San Diego, USA) and rabbit anti-Iba-1 (1:600; Wako, Osaka, Japan). Then retinas were incubated with the secondary antibodies: goat anti-rat α-IgG conjugated to Alexa Fluor 488 (1:150; Invitrogen, Paisley UK) and donkey anti-rabbit antibody conjugated to Alexa Fluor 594 (1:800; Invitrogen).

Two negative controls were also performed. In the first, there was no primary antibody, and the samples were incubated with the secondary antibody and the diluents used for the antibodies. In the second, the tissue was only incubated with the diluents used in the primary and secondary antibodies. This second control allowed assessment of how much endogenous fluorescence contributed to the observed fluorescence^74^.

Retinas were studied using a fluorescence microscope (Axioplan 2 Imaging Microscope, Carl Zeiss) to which an ApoTome device (Carl Zeiss, Munich, Germany) and a high-resolution digital camera (Cool-SNAP Photometrics, Tucson, AZ, USA) were associated. The microscope has a Zeiss 10 filter set for Alexa Fluor 488 and a Zeiss 64 filter set for Alexa Fluor 594. Using the motorized xyz microscope stage, the retinal whole-mounts were studied along the xyz axes. Cellular components located in the same xz plane were considered to be located in the same focal plane. Images were taken with the apotome device, which creates optical sections of fluorescent samples- free of scattered light and increases the resolution in Z direction compared to conventional fluorescence microscopy. Z-stacks were analyzed in Axiovision version 4.2 (Carl Zeiss). Adobe Photoshop CS3 Extended 10.0 program (Adobe Systems, San José, CA, USA) was used in the assembly of the figure plates.

### Quantitative retina analysis

To define the impact of OHT on Iba-1+ cells at the various time points, the following outcomes were quantified by an analyst blinded to the retina treatment: (a) the number of Iba-1+ cells cells in the OS, OPL and IPL; (b) Iba1-RA in the NFL-GCL; (c) the arbor area of the Iba-1+ cells in the OPL and IPL; (d) the number of microglial vertical processes connecting the OPL and OS; (e) the cell body area of Iba-1+ cells in the OPL, IPL and NFL-GCL; and (f) the mean of P2RY12 expression intensity. The different morphology that the microglial cells adopted in the different retinal layers (OS, OPL, IPL and NFL-GCL) allowed us to identify in which retinal layer we were located. In addition the weak fluorescence emitted by the somas of retinal nuclear layers and recorded with Zeiss filter set 10 for Alexa Fluor 488, also enabled the location of the retinal layer in the retinal whole-mount.

Quantification was carried in all naïve control eyes (n = 6) as well as all OHT and contralateral eyes of all animals in all the laser-treated groups as we described previously^32,65,75,76^. For the retinal count of Iba-1+ cells, in the retinal whole mounts, equivalent areas were photographed in the vertical and horizontal meridians, which crossed the optic nerve, and which included the upper, lower, nasal and temporal areas. To prevent a portion of the retinal whole-mount were being duplicated or omitted, all the fields analyzed were contiguous. Each meridian was analyzed using the motorized stage of the microscope to scan its entire length along the X-Y axis, giving an approximate total of 550 fields evaluated^32,75,76^. The photographs were made at 20x magnification, providing an area of 0.1502 mm^2^ per field. Besides, to quantify Iba-1+ cells in the OPL, IPL, and NFL-GCL, the entire preparation was studied along the Z axis in depth every 2 μm.

The method used for the Iba-1+ cell quantification depended on the retinal layer. In the plexiform layers, microglial cells were distributed throughout the retina forming a regular mosaic-shaped plexus, and therefore, these cells could be individually identified and counted automatically^75,77^. However, in the NFL-GCL the microglial cells cannot be so easily identified, especially in the HTO eyes, so in this layer the Iba1-RA was used for quantification^75,77^. In OS, Iba-1+ cells easily individualized, but did not form a regular plexus, which hindered their automatic counting, therefore, in this layer a manual counting was used.

### Iba-1+ cell number in the OS

In OS, the Iba-1 + cell count was performed using the “Interactive” manual counting tool in AxioVision 4.8.2 software (Carl Zeiss) that is associated with the Apotome device and the fluorescence microscope.

### Iba-1+ cell number in the OPL, IPL and NFL-GCL

In these retinal layers Iba-1+ cells were counted using an algorithm developed in MATLAB by our group^75–77^ that allows quantifying the number of Iba-1 + cells in the OPL and IPL and the Iba-1RA in the GCL-NFL. To quantify the number of Iba-1 + cells, we proceeded according to our protocol^75,77^. In summary, a Z-stack projection was performed on the selected images. The image was normalized to the pixel that had a higher value in the image, so that the image values were in a range between 0–1. The threshold was then imposed so that all values <0.2 were set to 0 and the remaining values were maintained. The remaining image was divided into segments and the center of mass of each segment was determined to identify the presence or absence of cells. To avoid counting twice the same cell that is between two adjacent segments, the minimum distance that separates the cells was specified. All points that are at a distance less than this minimum distance were considered to belong to the same cell, and therefore were counted only once.

For the quantification of Iba1-RA, we use the threshold tool in MATLAB in the selected images^75,77^. The thresholds determine the objects of interest by grayscale values and allow them to be differentiated from other areas of the image. After that, we quantify the Iba1-RA using the “count NFL” algorithm.

### Arbor area of Iba-1+ cells in the OPL and IPL

We studied four equivalent areas in each plexiform layer along the horizontal and vertical meridians that cross the optic nerve. To have a representation of the all retinal tissue, we select different retinal areas at various distances from the optic disc, in the different quadrants of the retina as follow: the retinal area nearby to the optic disc in the superior retina; the area at two levels of eccentricity in the inferior retina; the area at three levels of eccentricity in the nasal retina and the area at four levels of eccentricity in the temporal retina. In photographs taken at 20×, the arbor area of the Iba-1 + cells in the plexiform layers was quantified^32^. For it, a polygon was drawn that connected the most distal points of the Iba-1 + cellular processes using the” interactive Measurement” tool of the AxioVision software (Carl Zeiss).

### Number of vertical processes of Iba-1+ cells connecting the OPL and OS

To make this quantification, we took 20x photographs in the retinal plane between the OPL and the OS. The vertical processes of the Iba-1+ cells that connected both retinal layers were observed as points, which were quantified using the manual counting tool of the AxioVision software.

### Cell body area of Iba-1+ cells in the OPL, IPL and NFL-GCL

The cell body area was measured in the OPL, IPL and NFL-GCL using the same areas as those used for the arbor area. In photographs taken at 20×, the contours of the cell bodies were traced using the “interactive measurement” tool of the AxioVision software.

### Mean P2RY12 intensity expression

In the retinal whole mounts labelled with P2RY12 antibody, equivalent areas were photographed at 20x in the vertical and horizontal meridians, including the upper, lower, nasal and temporal areas in different retinal layers (OPL, IPL and NFL-GCL). The photographs were taken with equal values of time exposure and intensity of excitation. To quantify P2RY12 expression intensity, we used an algorithm developed in MATLAB (© MathWorks, Inc) and AxioVision 4.8.2 software (Carl Zeiss) that is associated with the Apotome device and the fluorescence microscope. The algorithm allows us to identify different intensity levels of P2RY12 expression in the OPL, IPL and NFL-GCL in the different groups of study. Image Average Intensity was calculated by adding the pixel values of the green channel (P2RY12 immunostaining) of each image and dividing this result by the total number of pixels of the image. The percentages we show in this paper are the result of normalizing the Image Average Intensity values by 255 that is the maximum value each pixel can represent.

### Statistical analysis

The SPSS 22 program (IBM, Chicago, IL, USA) was used to perform the statistics. Data were provided as mean ± SD. In the following parameters: (i) IOP; (ii) Iba-1+ cell number (OS, OPL and IPL); (iii) Iba1-RA (NFL-GCL); (iv) arbor area of Iba-1+ cells (OPL and IPL); (v) number of Iba-1+ vertical processes; (vi) cell body area of Iba-1+ cells (OPL, IPL and NFL-GCL), and vii) mean P2RY12 intensity expression, differences between naïve, OHT and contralateral eyes were analyzed using the Wilcoxon W test (for paired data) and Mann Whitney U test (for unpaired data). At the different time points, differences between contralateral and OHT eyes were evaluated using the ANOVA test with Bonferroni correction.

In the following parameters of OHT eyes: (i) number of Iba-1+ cells (OS, OPL and IPL) in all retinal zones; (ii) number of Iba-1+ cells (OS, OPL and IPL), taking into account only the zones nearest to the optic disc; (iii) Iba1-RA (NFL-GCL) in all retinal zones; and (iv) Iba1-RA (NFL-GCL), taking into account only the zones closest to the optic disc, the differences between several areas of the retina (superior, inferior, nasal and temporal) were analyzed using the ANOVA test with Bonferroni correction.

We consider that the differences were significant when p < 0.05.

## Supplementary information

Supplementary Information.

## References

[1] Quigley HA, Broman AT (2006). The number of people with glaucoma worldwide in 2010 and 2020. Br. J. Ophthalmol..

[2] Yang S (2018). Alpha 1-antitrypsin inhibits microglia activation and facilitates the survival of iPSC grafts in hypertension mouse model. Cell. Immunol..

[3] Agarwal R, Gupta SK, Agarwal P, Saxena R, Agrawal SS (2009). Current concepts in the pathophysiology of glaucoma. Indian J. Ophthalmol..

[4] Quigley HA (1995). Ganglion cell death in glaucoma: pathology recapitulates ontogeny. Aust. N. Z. J. Ophthalmol..

[5] Soto I, Howell GR (2014). The complex role of neuroinflammation in glaucoma. Cold Spring Harb. Perspect. Med..

[6] Grieshaber MC, Orgul S, Schoetzau A, Flammer J (2007). Relationship Between Retinal Glial Cell Activation in Glaucoma and Vascular Dysregulation. J. Glaucoma.

[7] Yanagi M (2011). Vascular risk factors in glaucoma: a review. Clin. Experiment. Ophthalmol..

[8] Tezel G (2010). Oxidative stress and the regulation of complement activation in human glaucoma. Invest. Ophthalmol. Vis. Sci..

[9] Gallego BI (2012). IOP induces upregulation of GFAP and MHC-II and microglia reactivity in mice retina contralateral to experimental glaucoma. J Neuroinflammation.

[10] Williams PA (2017). Neuroinflammation in glaucoma: A new opportunity. Exp. Eye Res..

[11] Gramlich OW (2016). Immune response after intermittent minimally invasive intraocular pressure elevations in an experimental animal model of glaucoma. J. Neuroinflammation.

[12] Bosco A (2008). Reduced retina microglial activation and improved optic nerve integrity with minocycline treatment in the DBA/2J mouse model of glaucoma. Invest. Ophthalmol. Vis. Sci..

[13] Levkovitch-Verbin H, Kalev-Landoy M, Habot-Wilner Z, Melamed S (2006). Minocycline delays death of retinal ganglion cells in experimental glaucoma and after optic nerve transection. Arch. Ophthalmol. (Chicago, Ill. 1960).

[14] Bosco A (2012). Early reduction of microglia activation by irradiation in a model of chronic glaucoma. PLoS One.

[15] Wang J-W, Liu Y-M, Zhao X-F, Zhang H (2017). Gastrodin protects retinal ganglion cells through inhibiting microglial-mediated neuroinflammation in an acute ocular hypertension model. Int. J. Ophthalmol..

[16] Langmann T (2007). Microglia activation in retinal degeneration. J. Leukoc. Biol..

[17] Ransohoff RM, Cardona AE (2010). The myeloid cells of the central nervous system parenchyma. Nature.

[18] Borkenstein A (2013). Measurement of tumor necrosis factor-alpha, interleukin-6, Fas ligand, interleukin-1α, and interleukin-1β in the aqueous humor of patients with open angle glaucoma using multiplex bead analysis. Mol. Vis..

[19] Madeira MH (2015). Contribution of microglia-mediated neuroinflammation to retinal degenerative diseases. Mediators Inflamm..

[20] Cueva Vargas JL (2015). Soluble Tumor Necrosis Factor Alpha Promotes Retinal Ganglion Cell Death in Glaucoma via Calcium-Permeable AMPA Receptor Activation. J. Neurosci..

[21] Liu X (2016). The Effect of A2A Receptor Antagonist on Microglial Activation in Experimental Glaucoma. Invest. Ophthalmol. Vis. Sci..

[22] Haynes SE (2006). The P2Y12 receptor regulates microglial activation by extracellular nucleotides. Nat. Neurosci..

[23] Davalos D (2005). ATP mediates rapid microglial response to local brain injury *in vivo*. Nat. Neurosci..

[24] Masuda T (2014). IRF8 is a transcriptional determinant for microglial motility. Purinergic Signal..

[25] Carbonell WS (2005). Migration of Perilesional Microglia after Focal Brain Injury and Modulation by CC Chemokine Receptor 5: An *In Situ* Time-Lapse Confocal Imaging Study. J. Neurosci..

[26] Zrzavy T (2018). Dominant role of microglial and macrophage innate immune responses in human ischemic infarcts. Brain Pathol..

[27] Butovsky O (2014). Identification of a unique TGF-β–dependent molecular and functional signature in microglia. Nat. Neurosci..

[28] Mildner A, Huang H, Radke J, Stenzel W, Priller J (2017). P2Y12 receptor is expressed on human microglia under physiological conditions throughout development and is sensitive to neuroinflammatory diseases. Glia.

[29] Sipe GO (2016). Microglial P2Y12 is necessary for synaptic plasticity in mouse visual cortex. Nat. Commun..

[30] Seitz R, Ohlmann A, Tamm ER (2013). The role of Müller glia and microglia in glaucoma. Cell Tissue Res..

[31] de Hoz R (2018). Bilateral early activation of retinal microglial cells in a mouse model of unilateral laser-induced experimental ocular hypertension. Exp. Eye Res..

[32] Rojas B (2014). Microglia in mouse retina contralateral to experimental glaucoma exhibit multiple signs of activation in all retinal layers. J. Neuroinflammation.

[33] de Hoz R (2017). Early signs of microglial activation in mice retinas contralateral to experimental glaucoma: quantitative analysis of cells number, processes retraction and reorientation. Acta Ophthalmol..

[34] Howell GR (2012). Radiation treatment inhibits monocyte entry into the optic nerve head and prevents neuronal damage in a mouse model of glaucoma. J. Clin. Invest..

[35] Yang F, Wu L, Guo X, Wang D, Li Y (2013). Improved retinal ganglion cell survival through retinal microglia suppression by a chinese herb extract, triptolide, in the DBA/2J mouse model of glaucoma. Ocul. Immunol. Inflamm..

[36] Inman DM, Horner PJ (2007). Reactive nonproliferative gliosis predominates in a chronic mouse model of glaucoma. Glia.

[37] Bosco A (2015). Neurodegeneration severity can be predicted from early microglia alterations monitored *in vivo* in a mouse model of chronic glaucoma. Dis. Model. Mech..

[38] Bosco, A., Romero, C. O., Ambati, B. K. & Vetter, M. L. *In vivo* dynamics of retinal microglial activation during neurodegeneration: confocal ophthalmoscopic imaging and cell morphometry in mouse glaucoma. *J. Vis. Exp*. e52731 (2015). 10.3791/52731 PMC4542682 25992962

[39] Wang X, Tay SS, Ng YK (2000). An immunohistochemical study of neuronal and glial cell reactions in retinae of rats with experimental glaucoma. Exp. brain Res..

[40] Naskar R, Wissing M, Thanos S (2002). Detection of early neuron degeneration and accompanying microglial responses in the retina of a rat model of glaucoma. Invest. Ophthalmol. Vis. Sci..

[41] Zhang C, Lam TT, Tso MO (2005). Heterogeneous populations of microglia/macrophages in the retina and their activation after retinal ischemia and reperfusion injury. Exp. Eye Res..

[42] Taylor S (2011). Involvement of the CD200 receptor complex in microglia activation in experimental glaucoma. Exp. Eye Res..

[43] Trost A (2015). Time-dependent retinal ganglion cell loss, microglial activation and blood-retina-barrier tightness in an acute model of ocular hypertension. Exp. Eye Res..

[44] Noristani R (2016). Retinal and Optic Nerve Damage is Associated with Early Glial Responses in an Experimental Autoimmune Glaucoma Model. J. Mol. Neurosci..

[45] Madeira MH (2016). Caffeine administration prevents retinal neuroinflammation and loss of retinal ganglion cells in an animal model of glaucoma. Sci. Rep..

[46] Brown GC, Neher JJ (2014). Microglial phagocytosis of live neurons. Nat. Rev. Neurosci..

[47] Ramirez AIAI (2017). The role of microglia in retinal neurodegeneration: Alzheimer’s disease, Parkinson, and glaucoma. Front. Aging Neurosci..

[48] De Hoz R (2013). Rod-like microglia are restricted to eyes with laser-induced ocular hypertension but absent from the microglial changes in the contralateral untreated eye. PLoS One.

[49] Yuan L, Neufeld AH (2001). Activated microglia in the human glaucomatous optic nerve head. J. Neurosci. Res..

[50] Johnson EC, Jia L, Cepurna WO, Doser TA, Morrison JC (2007). Global changes in optic nerve head gene expression after exposure to elevated intraocular pressure in a rat glaucoma model. Invest. Ophthalmol. Vis. Sci..

[51] Garden GA, Möller T (2006). Microglia biology in health and disease. J. Neuroimmune Pharmacol..

[52] Giulian D, Ingeman JE (1988). Colony-stimulating factors as promoters of ameboid microglia. J. Neurosci..

[53] Salinas-Navarro M (2010). Ocular hypertension impairs optic nerve axonal transport leading to progressive retinal ganglion cell degeneration. Exp. Eye Res..

[54] Vidal-Sanz M (2012). Understanding glaucomatous damage: anatomical and functional data from ocular hypertensive rodent retinas. Prog. Retin. Eye Res..

[55] Shapiro LA, Perez ZD, Foresti ML, Arisi GM, Ribak CE (2009). Morphological and ultrastructural features of Iba1-immunolabeled microglial cells in the hippocampal dentate gyrus. Brain Res..

[56] Okunuki Y (2018). Microglia inhibit photoreceptor cell death and regulate immune cell infiltration in response to retinal detachment. Proc. Natl. Acad. Sci. USA.

[57] Ma W, Zhao L, Fontainhas AM, Fariss RN, Wong WT (2009). Microglia in the mouse retina alter the structure and function of retinal pigmented epithelial cells: a potential cellular interaction relevant to AMD. PLoS One.

[58] Xu H, Chen M, Forrester JV (2009). Para-inflammation in the aging retina. Prog. Retin. Eye Res..

[59] Combadière C (2007). CX3CR1-dependent subretinal microglia cell accumulation is associated with cardinal features of age-related macular degeneration. J. Clin. Invest..

[60] Salinas-Navarro M (2009). Functional and morphological effects of laser-induced ocular hypertension in retinas of adult albino Swiss mice. Mol. Vis..

[61] Cuenca N (2010). Changes in the inner and outer retinal layers after acute increase of the intraocular pressure in adult albino Swiss mice. Exp. Eye Res..

[62] Ortín-Martínez A (2015). Laser-induced ocular hypertension in adult rats does not affect non-RGC neurons in the ganglion cell layer but results in protracted severe loss of cone-photoreceptors. Exp. Eye Res..

[63] Wu L-J, Vadakkan KI, Zhuo M (2007). ATP-induced chemotaxis of microglial processes requires P2Y receptor-activated initiation of outward potassium currents. Glia.

[64] Ohsawa K (2007). Involvement of P2X4 and P2Y12 receptors in ATP-induced microglial chemotaxis. Glia.

[65] Koizumi S (2007). UDP acting at P2Y6 receptors is a mediator of microglial phagocytosis. Nature.

[66] Wang K, Peng B, Lin B (2014). Fractalkine receptor regulates microglial neurotoxicity in an experimental mouse glaucoma model. Glia.

[67] Gao H-M (2011). HMGB1 acts on microglia Mac1 to mediate chronic neuroinflammation that drives progressive neurodegeneration. J. Neurosci..

[68] Ramírez AI (2015). Macro-and microglial responses in the fellow eyes contralateral to glaucomatous eyes. Prog. Brain Res..

[69] Quigley HA, Hohman RM (1983). Laser energy levels for trabecular meshwork damage in the primate eye. Invest. Ophthalmol. Vis. Sci..

[70] Neufeld AH (1999). Microglia in the optic nerve head and the region of parapapillary chorioretinal atrophy in glaucoma. Arch. Ophthalmol. (Chicago, Ill. 1960).

[71] Shimazawa M, Yamashima T, Agarwal N, Hara H (2005). Neuroprotective effects of minocycline against *in vitro* and *in vivo* retinal ganglion cell damage. Brain Res..

[72] Triviño A, Ramírez JM, Ramírez AI, Salazar JJ, García-Sanchez J (1992). Retinal perivascular astroglia: an immunoperoxidase study. Vision Res..

[73] Butovsky O (2015). Targeting miR-155 restores abnormal microglia and attenuates disease in SOD1 mice. Ann. Neurol..

[74] Triviño A (2002). Distribution and organization of the nerve fiber and ganglion cells of the human choroid. Anat. Embryol. (Berl)..

[75] Gallego BI, de Gracia P (2016). Automatic counting of microglial cell activation and its applications. Neural Regen. Res..

[76] Fernández-Albarral JA (2019). Neuroprotective and Anti-Inflammatory Effects of a Hydrophilic Saffron Extract in a Model of Glaucoma. Int. J. Mol. Sci..

[77] de Gracia P (2015). Automatic Counting of Microglial Cells in Healthy and Glaucomatous Mouse Retinas. PLoS One.

