# Supplementary material for: Time course of bilateral microglial activation in a mouse model of laser-induced glaucoma
Source: Sci Rep. 2020 Mar 17;10:4890. doi: 10.1038/s41598-020-61848-9 (PMC7078298; doi:10.1038/s41598-020-61848-9)
Supplement: Supplementary file 1 — Supplementary Information. [file 41598_2020_61848_MOESM1_ESM.docx]

**Supplemental information**

**Time course of bilateral microglial activation in a mouse model of laser-induced glaucoma**

Ana I. Ramírez^1,2,#^, Rosa de Hoz^1,2,#^, José A. Fernandez-Albarral^1^, Elena Salobrar-Garcia^1,3^, Blanca Rojas^1,3^, Francisco J. Valiente-Soriano^4^, Marcelino Avilés-Trigueros^4^, María P. Villegas-Pérez^4^, Manuel Vidal-Sanz^4^, Alberto Triviño^1,3^, José M. Ramírez^1,3,^*, Juan J. Salazar^1,2,^*

^1^Instituto de Investigaciones Oftalmológicas Ramón Castroviejo. Universidad Complutense de Madrid. Spain.

^2^Facultad de Óptica y Optometría. Departamento de Inmunología, Oftalmología y ORL. Universidad Complutense de Madrid. Spain.

^3^Facultad de Medicina. Departamento de Inmunología, Oftalmología y ORL. Universidad Complutense de Madrid. Spain

^4^Departamento de Oftalmología, Facultad de Medicina, Universidad de Murcia and Instituto Murciano de Investigación Biosanitaria Virgen de la Arrixaca. Murcia, Spain

^#^These authors contributed equally to this work

*Corresponding authors:

José M. Ramírez ([ramirezs@med.ucm.es](mailto:ramirezs@med.ucm.es)), and Juan J. Salazar ([jjsalazar@med.ucm.es](mailto:jjsalazar@med.ucm.es))

Instituto de Investigaciones Oftalmológicas Ramón Castroviejo.

Facultad de Medicina, Pab 6, 4ª planta. Ciudad Universitaria (UCM). 28040. Madrid. Spain.


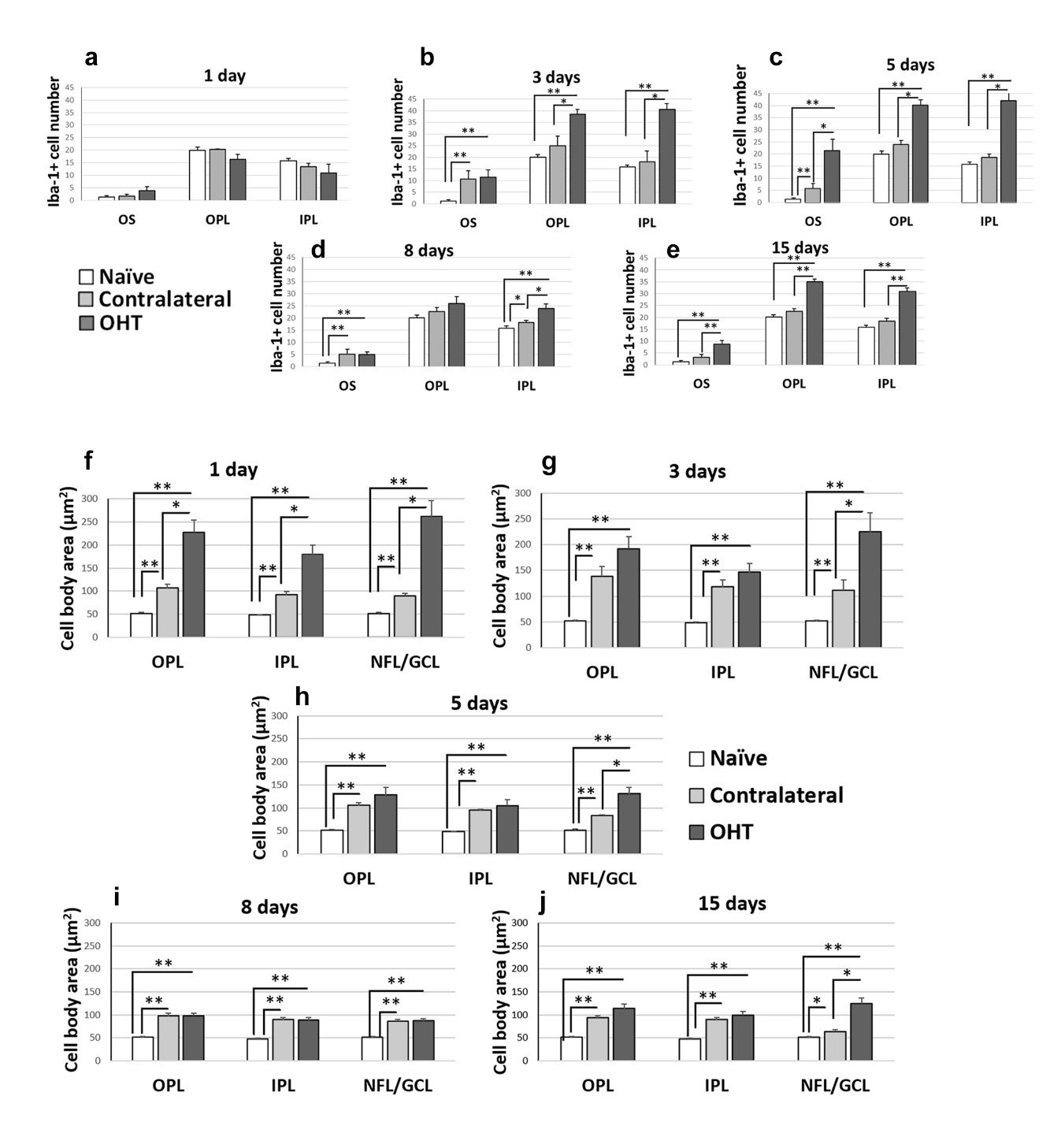


**Supplementary Figure S1.** **Iba-1+ cells in the photoreceptor outer segment layer (OS), outer plexiform layer (OPL), and the inner plexiform layer (IPL) as well as the cell body area of Iba-1 + cells in plexiform layers and nerve fiber-ganglion cell layer (NFL-GCL) at different times after laser-induced ocular hypertension (OHT)**.

**a-e:** Quantitative analysis of the Iba-1+ cell number in OS, OPL and IPL. Histograms show the mean (±SD) number of Iba-1+ cells per area of 0.1502 mm^2^ in naïve, contralateral and OHT eyes at 1 d (a), 3 d (b), 5 d (c), 8 d (d) and 15 d (e). **f-j:** Quantitative analysis of the cell body area of Iba-1+ cells in OPL, IPL and NFL-GCL. Histograms show a mean (±SD) cell body area of Iba-1+ cells in naïve, contralateral and OHT eyes at 1 d (f), 3 d (g), 5 d (h), 8 d (i) and 15 d (j). *p < 0.05, **p < 0.01


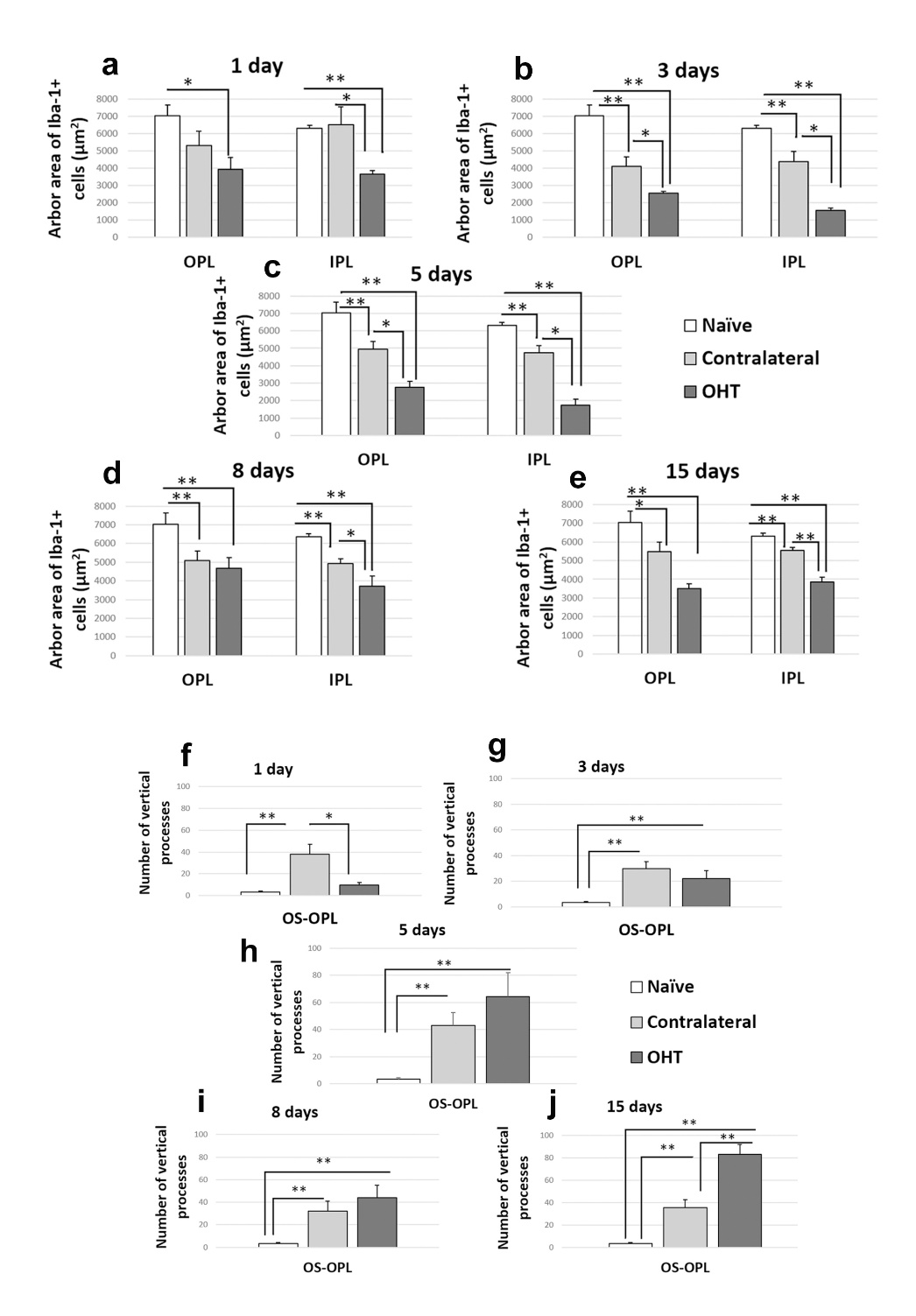
**Suplementary Figure S2. Arbor area (AA) of Iba-1 + cells in the outer plexiform layer (OPL) and inner plexiform layer (IPL) as well as Iba-1+ vertical processes (VP) between outer plexiform layer (OPL) and the photoreceptor outer segment layer (OS) at different times after laser-induced ocular hypertension (OHT).**

**a-e:** Quantitative analysis of the AA in OPL and IPL. Histograms show a mean AA (±SD) in naïve, contralateral and OHT eyes at 1 d (a), 3 d (b), 5 d (c), 8 d (d) and 15 d (e).

**f-j:** Quantitative analysis of the number of VP between OPL and OS. Histograms show mean number of VP (±SD) in naïve, contralateral and OHT eyes at 1 d (f), 3 d (g), 5 d (h), 8 d (i) and 15 d (j). *p < 0.05, **p < 0.01


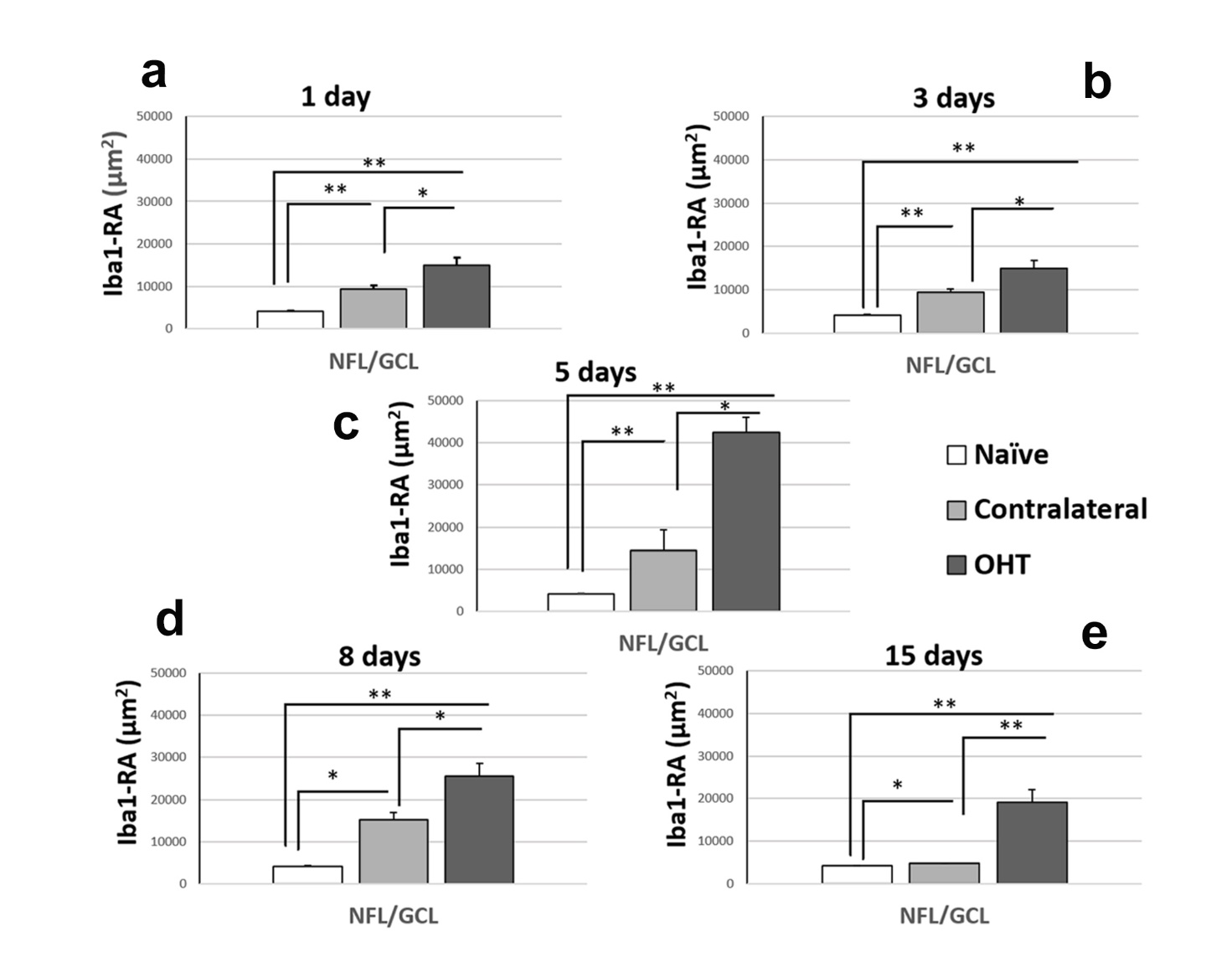


**Suplementary Figure S3. Retinal area occupied by Iba-1+ cells (Iba1-RA) in the nerve fiber-ganglion cell layer (NFL-GCL)**

Quantitative analysis of the Iba1-RA in NFL-GCL. Histograms show a mean Iba1-RA (±SD) for naïve, contralateral and OHT eyes at 1 d (a), 3 d (b), 5 d (c), 8 d (d) and 15 d (e). *p < 0.05, **p < 0.01.


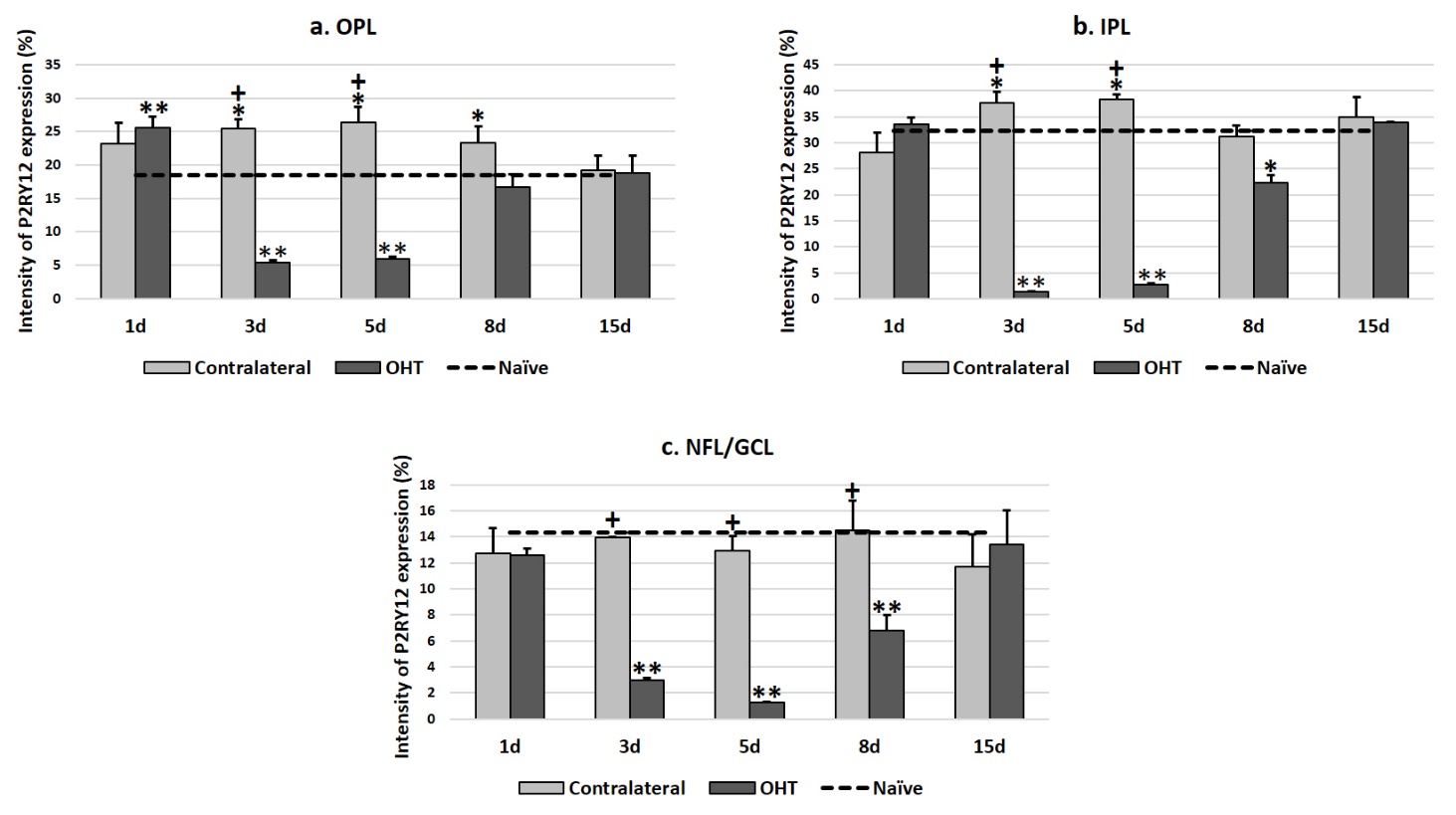


**Suplementary Figure S4. Intensity of P2RY12 expression in Iba-1+ cells in the outer plexiform layer (OPL), inner plexiform layer (IPL) and in the nerve fiber-ganglion cell layer (NFL-GCL)**

Quantification of the P2RY12 expression intensity over time in OPL (a), IPL (b) and NFL-GCL (c). Histograms show a mean P2RY12 expression intensity (±SD), expressed as a percentage, for naïve, contralateral and OHT eyes. *p < 0.05, **p < 0.01 vs Naïve; ^+^p < 0.05 vs OHT.
